# Association of mitochondrial phosphoenolpyruvate carboxykinase with prognosis and immune regulation in hepatocellular carcinoma

**DOI:** 10.1038/s41598-024-64907-7

**Published:** 2024-06-18

**Authors:** Chenxuan Li, En-di Zhang, Youzhi Ye, Zhongyun Xiao, Hanfei Huang, Zhong Zeng

**Affiliations:** 1 Kunming Medical University, Kunming, China; 2 The First Affiliated Hospital of Kunming Medical University, Kunming Medical University, Kunming, China

**Keywords:** PCK2, Prognosis, Immune infiltrates, Immune checkpoints, Epithelial–mesenchymal transition, Hepatocellular carcinoma, Gastrointestinal cancer, Cancer, Immunology, Biomarkers, Oncology

## Abstract

Mitochondrial phosphoenolpyruvate carboxykinase (PCK2), a mitochondrial isoenzyme, supports the growth of cancer cells under glucose deficiency conditions in vitro. This study investigated the role and potential mechanism of PCK2 in the occurrence and development of Hepatocellular carcinoma (HCC). The Cancer Genome Atlas (TCGA), Gene Expression Omnibus (GEO), and other databases distinguish the expression of PCK2 and verified by qRT-PCR and Western blotting. Kaplan–Meier was conducted to assess PCK2 survival in HCC. The potential biological function of PCK2 was verified by enrichment analysis and gene set enrichment analysis (GSEA). The correlation between PCK2 expression and immune invasion and checkpoint was found by utilizing Tumor Immune Estimation Resource (TIMER). Lastly, the effects of PCK2 on the proliferation and metastasis of hepatocellular carcinoma cells were evaluated by cell tests, and the expressions of Epithelial mesenchymal transformation (EMT) and apoptosis related proteins were detected. PCK2 is down-regulated in HCC, indicating a poor prognosis. PCK2 gene mutation accounted for 1.3% of HCC. Functional enrichment analysis indicated the potential of PCK2 as a metabolism-related therapeutic target. Subsequently, we identified several signaling pathways related to the biological function of PCK2. The involvement of PCK2 in immune regulation was verified and key immune checkpoints were predicted. Ultimately, after PCK2 knockdown, cell proliferation and migration were significantly increased, and N-cadherin and vimentin expression were increased. PCK2 has been implicated in immune regulation, proliferation, and metastasis of hepatocellular carcinoma, and is emerging as a novel predictive biomarker and metabolic-related clinical target.

## Introduction

Primary liver cancer, one of the most common and deadly malignant cancers in the world, is the sixth most common type of malignant tumor worldwide and the third most common cause of cancer death. By 2020, there were approximately 906,000 new cases and 830,000 deaths, with hepatocellular carcinoma accounting for 75–85%^1^. Up to now, the treatment of HCC has primarily comprised surgical resection and liver transplantation in the early stage, as well as various targeted and local therapies for advanced or inoperable patients^2^. Nonetheless, the prognosis and survival rate of patients remain unsatisfactory due to a number of issues including medication resistance, adverse effects, recurrence, and development of the disease^3^. Hence, the necessity for us to actively seek out novel therapeutic targets and tumour biomarkers for the diagnosis and treatment of HCC is growing.

A multi-step, multi-level process known as "tumorigenesis" occurs when certain cancer cells have evolved to multiply via metabolism in the presence of dietary limitation. By using phosphoenolpyruvate carboxykinase (PEPCK) to counteract glycolysis in cancer during glucose shortage, the reverse glycolysis pathway known as "gluconeogenesis" is activated^4^. PCK genes comprising cytoplasm (PCPEK-C or PCK1) and mitochondria (PEPCK-M or PCK2) are expressed in mammalian cells. Recent research has revealed that phosphoenolpyruvate carboxykinase (PEPCK) plays multiple roles in signal transduction, multiplication, and cancer stem cell (CSC) tumor phenotypes in addition to metabolic regulation^5^. Driven PCK1 expression in glucose-starved liver cancer cells has been shown in studies to induce tricarboxylic acid cycle (TCA) cataplerosis, resulting in an energy deficiency and oxidative stress, whereas supplementation of ketoglutarate or suppression of reactive oxygen species can prevent cell death provoked by PEPCK expression^6^. Whilst PCK1 has gained growing attention, PCK2's essence in HCC gluconeogenesis, metabolic reprogramming, cancer cell plasticity, and tumor progression should not be overlooked^7^. PCK2 is a phosphoenolpyruvate carboxykinase found in mitochondria that stimulates the production of phosphoenolpyruvate (PEP) when glucose levels are low. PEPCK is essential for tumor cell proliferation and tumor progression in vitro under low glucose circumstances^8^. Some human cancers have been reported to have a high level of PCK2 expression^9–11^, but it is in hepatocellular carcinoma that PCK2 expression is down-regulated, and low PCK2 expression is likely connected with a bad prognosis in HCC patients. In addition, whole-exome sequencing identified that PCK2 was associated with cancer cell proliferation in patients with hepatocellular carcinoma^12^, but there are few further studies on the molecular mechanism of PCK2, especially its correlation with clinical prognosis, metastasis, and immunity, which needs to be further confirmed.

In our investigation, a number of bioinformatics datasets, including TCGA, CPTAC, and HPA, were utilized to prove the discrepancies in the expression of PCK2 in hepatocellular carcinoma tissues and normal adjacent tissues at different levels. Survival and prognosis of PCK2 in HCC were analyzed using Kaplan–Meier plotter. Low PCK2 is an indicator of poor prognosis. The cBioPortal and Catalogue of Somatic Mutations in Cancer databases were used to assess PCK2 mutations in HCC. Furthermore, GO, KEGG, and GSEA analyses were used to verify the potential biological function of PCK2. Meanwhile, the Timer2.0 database was made to investigate the correction between PCK2 and immune cell infiltration in HCC, as well as the corresponding biomarkers, and to predict the key immune checkpoint. Finally, we conducted a series of cell experiments to verify the effects of PCK2 on the biological functions of hepatocellular cancer cell proliferation, invasion and migration, and also confirmed the relationship between PCK2 and EMT-related proteins and apoptoses-related proteins. Therefore, the in-depth study of PCK2 in hepatocellular carcinoma will provide a new scientific basis and possibility for the treatment and prognosis of hepatocellular carcinoma.

## Materials and methods

### Comparison of the PCK2 expression level

The Cancer Genome Atlas (TCGA) (https://genomecancer.ucsc.edu/) is a large, free of Cancer research reference database, which collect and organize the omics data of various kinds of Cancer. The TCGA (TCGA-HCC, n = 424) tools cancer browser was used to obtain data from HCC patients with RNA-Seq expression and pair clinical pathologic information. Clinical Proteomic Tumor Analysis Consortium (CPTAC) (http://ualcan.path.uab.edu/analysis-prot.html) merges genomic and proteomic information to discover all proteins in tumors and normal tissues that could be candidate biomarkers for cancer. CPTAC database was utilized to examine PCK2 protein expression levels in HCC and its normal tissues. The Human Protein Atlas (HPA) database (https://www.proteinatlas.org/) was made to collect immunohistochemical images of PCK2. From three dimensions of cell, tissue, and pathology, the HPA database brought to light the expression of proteins among cells, normal tissues, and malignant tissues.

### Kaplan–Meier plotter

By combining gene expression with clinical prognostic value and survival-related research, discovery, and reassurance of molecular markers, the Kaplan and Meier Plotter (http://kmplot.com/analysis/) information database tested the 21 types of cancer genes that affect survival. It was utilized to investigate the link between PCK2 expression and patient survival, including OS (overall survival), DSS (disease-specific survival), DFS (disease-free survival), and PFS (progression-free survival).

### Gene Expression Omnibus (GEO) and International Cancer Genome Consortium (ICGC)

To examine PCK2 transcription levels, we acquired RNA sequencing data from the Gene Expression Omnibus (GEO) (www.ncbi.nlm.nih.gov/geo) database. (GSE112790, n = 198; GSE102079, n = 257; GSE14520, n = 221). And in the ICGC database (https://dcc.icgc.org/), Survival analysis was performed in both data sets using R software (4.1.3) and the Survival package, and KM curves were plotted using the ggsurvplot function.

### Mutation analysis

The cBioPortal (http://www.cbioportal.org/) was used to assess the mutation frequency of PCK2 in HCC. The Catalogue of Somatic Mutations in Cancer (COSMIC) database (http://cancer.sanger.ac.uk) was used to further verify the mutation types of PCK2 in HCC.

### Tumor immune estimation resource (TIMER)

Tumor immune estimation resource (TIMER) (www.cistrome.shinyapps.io) is a trustworthy and user-friendly resource that contains gene expression profiles from the TCGA. The TIMER approach can be used to assess immune cell infiltration and its therapeutic potential. We explored the correlation between PCK2 and multiple linked tumor immune cell encroachment, the cumulative survival between rising and falling expression levels and multiple immune cell invasions, and the clinical impact of a correlation coefficient with both PCK2 and immune checkpoints obtained from the GEO database. *p < 0.05; **p < 0.01; ***p < 0.001; ****p < 0.0001. The significance of two groups of samples was tested by the Wilcox test, and the significance of three groups or more samples was tested by the Kruskal–Wallis test.

### Gene ontology (GO) analysis, Kyoto encyclopedia of genes and genomes (KEGG) analyses, and gene set enrichment analysis (GSEA)

We obtained PCK2 functional enrichment through GO and KEGG databases^13,14^. GO analysis and KEGG analysis were performed using R software (Veersion 4.3.0), clusterProfiler package (Version 4.8.1), and org.Hs.eg.db package (3.17.0). Based on the c2.all.v2023.1.Hs. entrez dataset, we analyzed the GSEA enrichment of PCK2 coexpressed genes using the clusterProfiler package, and visualized the results.

### Quantitative real-time PCR (RT-qPCR).

qRT-PCR was performed to verify gene expression at the RNA level. Primers used were PCK2, 5′-GGCTGAGAATACTGCCACACT-3′ (forward), 5′-ACCGTCTTGCTCTCTACTCGT-3′ (reverse); GAPDH, 5′-CAGGAGGCATTGCTGATGAT-3′ (forward), 5′-GAAGGCTGGGGCTCATTT-3′ (reverse). GAPDH served as the reference gene since it showed stable expression over all samples. Melting curves were analyzed to verify the correct amplification of the expected RT-qPCR product.

### Western blot assay

Cells or tissues were cultured on ice for 0.5 h with RIPA buffer with 1 mM PMSF buffer (Solebo, China) and protease inhibition cocktail (Solebo, China). Protein concentration was determined by BCA protein analysis kit (Biyuntian, China). They were separated by 10% SDS-PAGE and transferred to PVDF membrane. The membrane was TBST treated in 5% skimmed milk for 120 min and incubated at 4 °C with specific primary antibodies including E-cadherin (1:20,000, 20874-1-AP, Proteintech), N-cadherin (1:8000, 22018-1-AP, Proteintech), Vimentin (1:5000, 10366-1-AP, Proteintech), Bcl2 (1:2000, 26593-1-AP, Proteintech), Bax (1:4000, 50599-2-Ig, Proteintech), PCK2 (1:10,000, ab187145, Abcam) and GAPDH (1:10,000, 60004-1-Ig, Proteintech) at 4 °C. The membrane was then cleaned with TBST and incubated with goat anti-mouse or goat anti-rabbit fluorescent conjugate secondary antibody for 60 min. After incubation, PVDF membranes were cleaned and visualized. ImageJ software was utilized to analyze the densitometric.

### Human HCC tissue specimens

We collected fresh and paracancer clinical samples from 8 HCC patients in the First Affiliated Hospital of Kunming Medical University. All of these patients underwent histopathological examination. The sample was approved by the Clinical Ethics Committee of the First Affiliated Hospital of Kunming Medical University and the patient's written informed consent was obtained. Clinical samples were used for subsequent RT-qPCR, western blot and other experiments.

### Cell culture and transfection

HCC cell lines (HepG2, Huh7, LM3 and Sk-Hep1) were purchased. HCC cells were cultured in DMEM medium with 10% fetal bovine serum (TransGen Biotech) and 1% antibiotics, and incubated at 37 °C in 5% CO_2_. Purchase plasmid with low PCK2 knockdown. The sequences of sh-PCK2-2 and sh-PCK2-3 were 5′-TGCTTGGATGAGGTTTGACAGTGAA-3′ and 5′-CATCAACCCTGAGAACGGCTTCTTT-3′. HCC cells were transfected with PolyFast (MCE). PCK2 overexpressed viruses were purchased and transfected according to the instructions. And the corresponding empty vector transfection HCC cells.

### EdU assay

A total of 300,000 cells were spread onto a slipper in a 12-well plate. The cells were then maintained with the prepared EdU working solution for 2 h. The cells were fixed with 4% paraformaldehyde and treated with 0.3%Triton X-100 for 15 min. The reaction solution in EdU cell proliferation detection was incubated for 30 min away from light. After washing, the cells were stained with DAPI and photographed under fluorescence microscope.

### Wound-healing assays

The transfected hepatoma cells were placed in 6-well plates. At 90% confluence, the cells were cleaned twice with PBS and marked with standard 200 μL pipette. The cells were regrown in culture without fetal bovine serum, and the extent of the wound was monitored and imaged using a microscope.

### Transwell migration assays

In the transwell cell chamber (Falcon, USA) of the 24-well plate, 200,000 hepatoma cells were inoculated. The cells on the top surface of the chamber were wiped away after 24 h of incubation at 37 °C. Cells on the cavity's bottom surface were fixed with 4% paraformaldehyde for 15 min and stained with crystal violet (Beyotime, China) for 10 min. A positive microscope was used to image and count the number of migrating cells.

### Statistical analysis

The results were displayed with P-values based on a log-rank test and a hazard ratio (HR). Spearman's correlation coefficients and P-values were used to evaluate gene co-relation. P-values less than 0.05 were saw as statistically significant.

## Results

### Lower PCK2 expression in tumor samples than that in normal tissues

**Figure 1:**
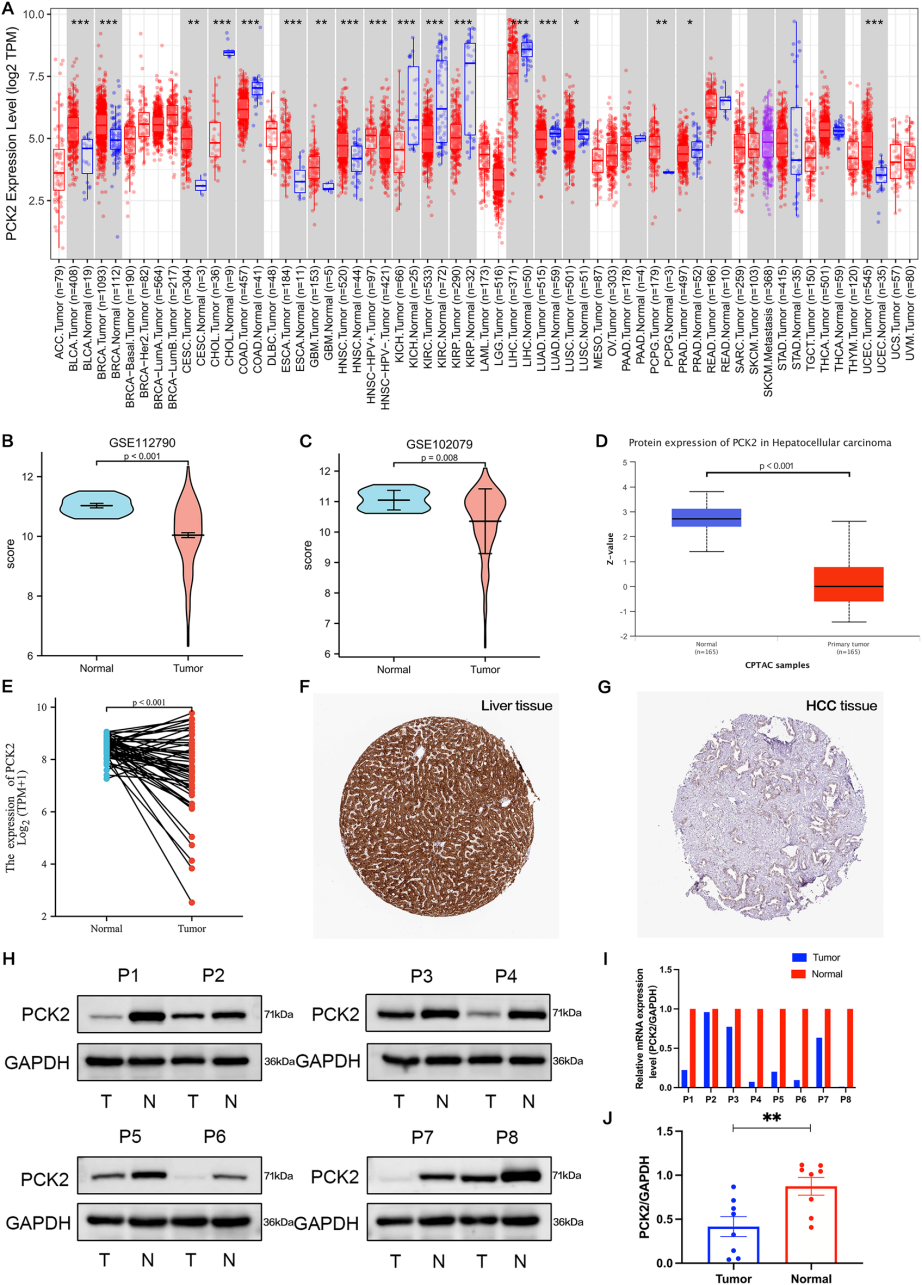
Hepatocellular carcinoma (HCC) and pan-carcinoma expression of PCK2. TIMER was used to measure PCK2 expression levels in various tumor types (**A**). The GSE112790 data set was utilized to compare the levels of PCK2 expression in HCC versus adjacent normal tissues (**B**). The GSE102079 data set was used to compare PCK2 expression levels in HCC and surrounding normal tissues (**C**). The CPTAC dataset was used to look at the level of PCK2 protein expression in normal tissues and adjacent normal tissues (**D**). A contrast of PCK2 expression levels in HCC and adjacent normal tissues using the TCGA dataset (**E**). The quantity of PCK2 protein in HCC tissue was fairly low than in adjacent normal tissue in the Human Protein Atlas (Antibody HPA051162, 10×) (**F**,**G**). Western blotting was used to detect PCK2 protein levels in 8 pairs of HCC tissues and their adjacent normal tissues (**H**,**J**). The mRNA level of PCK2 in 8 pairs of HCC tissues and their paired normal adjacent tissues(**I**).

To explore the possibility of PCK2, we used the TIMER database for analysis. The expression of PCK2 is different in a series of cancers and adjacent tissues. PCK2 was highly expressed in seven cancer types, including bladder urothelial carcinoma (BLCA), breast invasive carcinoma (BRCA), cervical and endocervical cancer (CESC), esophageal carcinoma (ESCA), glioblastoma multiforme (GBM), head and neck squamous cell carcinoma (HNSC), pheochrocytoma and paraganglioma (PCPG). However, expression was strongly down-regulated in nine of these cancer types, including cholangiocarcinoma (CHOL), colon adenocarcinoma (COAD), kidney chromophobe (KICH), kidney renal clear cell carcinoma (KIRC), kidney renal papillary cell carcinoma (KIRP), liver hepatocellular carcinoma (LIHC), lung adenocarcinoma (LUAD), lung squamous cell carcinoma (LUSC), and prostate adenocarcinoma (PRAD) (Fig. 1A). However, the PCK2 gene has been less studied in LIHC, so we collected a total of 455 samples from the GSE112790 and GSE102079 datasets, including 29 normal tissues and 426 liver cancer tissues. The expression of PCK2 mRNA in HCC was lower than that in adjacent normal tissues (P < 0.001) (Fig. 1B,C), which was also verified from the TCGA database (P < 0.001) (Fig. 1E). Further comparison of PCK2 protein expression levels based on the CPTAC database showed that PCK2 protein expression in HCC was substantially lower than that in adjacent normal tissues (Fig. 1D). Further, it was verified in the HPA database (Antibody HPA051162,10×) (Fig. 1F,G). Meanwhile, eight pairs of hepatocellular carcinoma and its adjacent paired samples were collected clinically, and the low expression of PCK2 in HCC tissues was confirmed by Western blotting (Fig. 1H,J) and qRT-PCR (Fig. 1I) through three repeated experiments. These results all prove that PCK2 is down-regulated in HCC.

### PCK2 expression correlated with clinical characteristics and survival analysis

**Figure 2:**
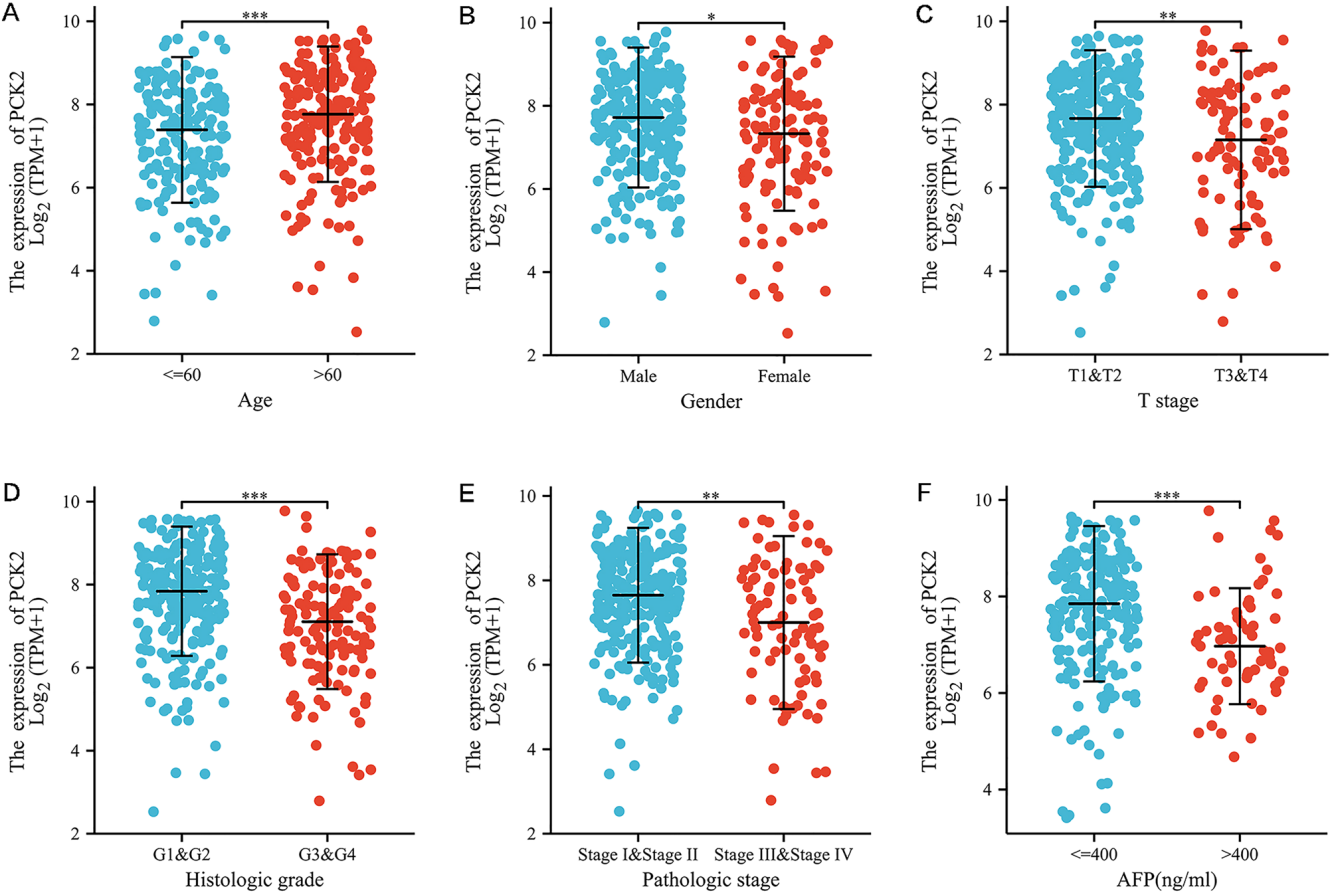
Dot plot assessing PCK2 expression in HCC patients depending on the clinical characteristics. Age (**A**). Gender (**B**). T stage (**C**). Histological grade (**D**). Pathological stage (**E**). AFP (**F**). Graphs are gerived from the TCGA database. *P < 0.05; **P < 0.01; ***P < 0.001.

The TCGA database was used to glean data on 374 patients with hepatocellular carcinoma, including clinical and gene expression data. Based on the mean value of PCK2 expression, patients were classified into high and low PCK2 expression groups, and possible linkages between PCK2 expression and clinical features were taken into account using the Wilcoxon signed-rank test and logistic regression analysis (Table 1). Analysis showed that the expression of PCK2 was significantly different from the TNM stage, pathological stage, and histological grade (P < 0.05). Patients at more advanced cancer stages tended to have lower expression levels of PCK2, and the expression of PCK2 was also tied to age, gender, and AFP (Alpha-fetoprotein) level (Fig. 2).

**Table 1:** Correlations between the expression of PCK2 and clinicopathologic characteristics in HCC.

Characteristic	Low expression of PCK2	High expression of PCK2	P
N	187	187	
T stage, n (%)			**0.025**
T1	81 (21.8%)	102 (27.5%)	
T2	50 (13.5%)	45 (12.1%)	
T3	51 (13.7%)	29 (7.8%)	
T4	5 (1.3%)	8 (2.2%)	
Pathologic stage, n (%)			0.050
Stage I	79 (22.6%)	94 (26.9%)	
Stage II	45 (12.9%)	42 (12%)	
Stage III	54 (15.4%)	31 (8.9%)	
Stage IV	3 (0.9%)	2 (0.6%)	
Gender, n (%)			**0.047**
Female	70 (18.7%)	51 (13.6%)	
Male	117 (31.3%)	136 (36.4%)	
Age, n (%)			**0.006**
≤ 60	102 (27.3%)	75 (20.1%)	
> 60	84 (22.5%)	112 (30%)	
Age, meidan (IQR)	59 (48, 67.75)	64 (55, 70)	** < 0.001**
Weight, n (%)			**0.002**
≤ 70	106 (30.6%)	78 (22.5%)	
> 70	66 (19.1%)	96 (27.7%)	
BMI, n (%)			**0.004**
≤ 25	103 (30.6%)	74 (22%)	
> 25	67 (19.9%)	93 (27.6%)	
Histologic grade, n (%)			** < 0.001**
G1	16 (4.3%)	39 (10.6%)	
G2	79 (21.4%)	99 (26.8%)	
G3	84 (22.8%)	40 (10.8%)	
G4	7 (1.9%)	5 (1.4%)	
AFP(ng/ml), n (%)			** < 0.001**
≤ 400	89 (31.8%)	126 (45%)	
> 400	49 (17.5%)	16 (5.7%)	

Significant values are in bold.

**Figure 3:**
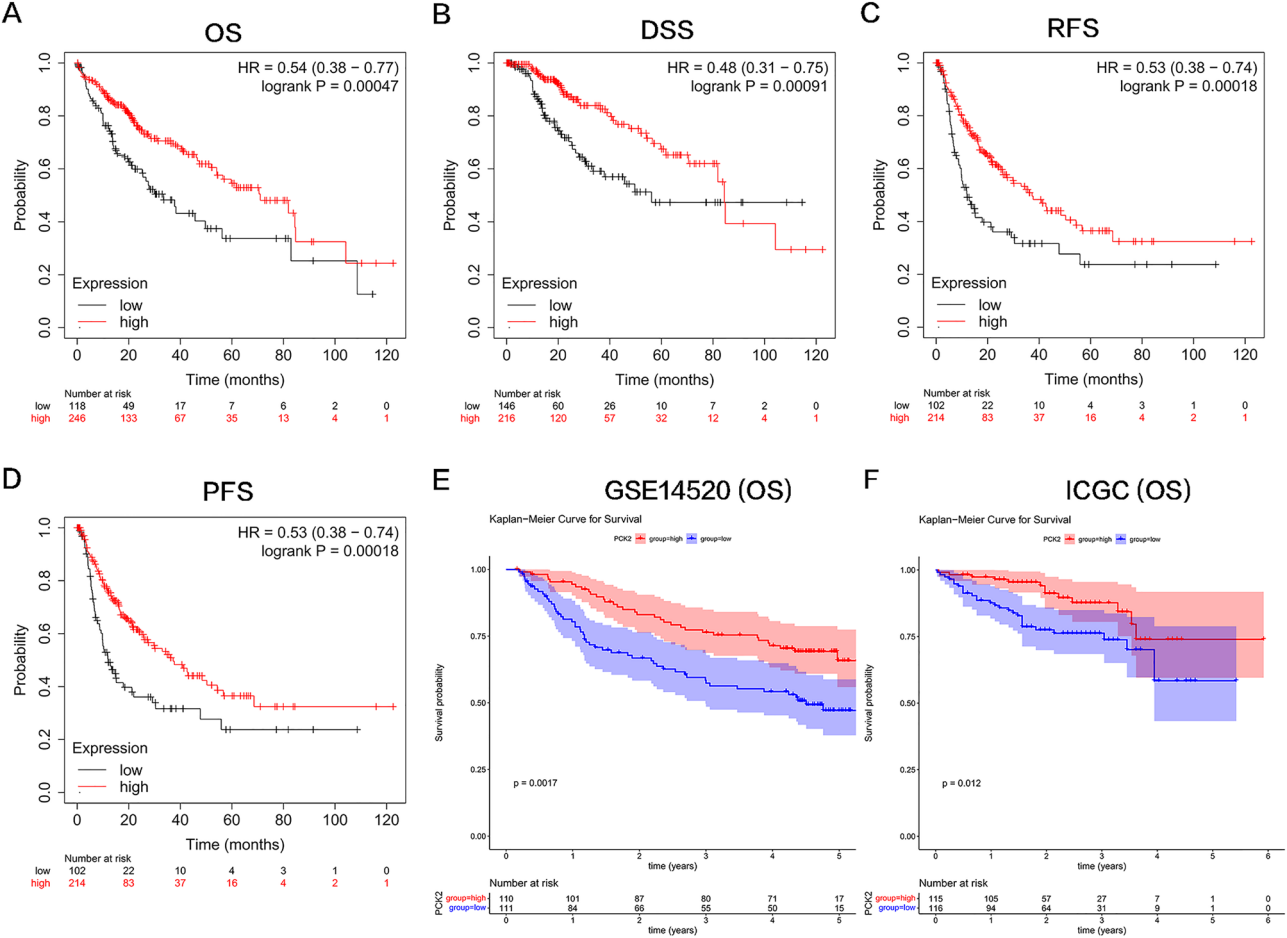
PCK2 expression is associated with a bad outcome in HCC. The prognostic values of PCK2 in HCC patients are seen in graphs derived from the Kaplan–Meier Plotter database (**A**-**D**). OS curve of PCK2 in GSE14520 and ICGC (**E**–**F**). *OS* overall survival, *RFS* relapse-free survival, *PFS* progression-free survival, *DSS* disease-specific survival, *HR* hazard ratio.

In addition, Kaplan–Meier survival analysis revealed that decreased PCK2 expression was significantly associated with poor overall survival, relapse-free survival, progression-free survival, and disease-specific survival in patients (Fig. 3A–D, P < 0.001) and was further verified by ICGC database (LIRI-JP) and GEO database (GSE14520) (Fig. 3E,F). In conclusion, low PCK2 expression was linked to a poor prognosis in patients with HCC.

### Mutations of PCK2 in HCC

**Figure 4:**
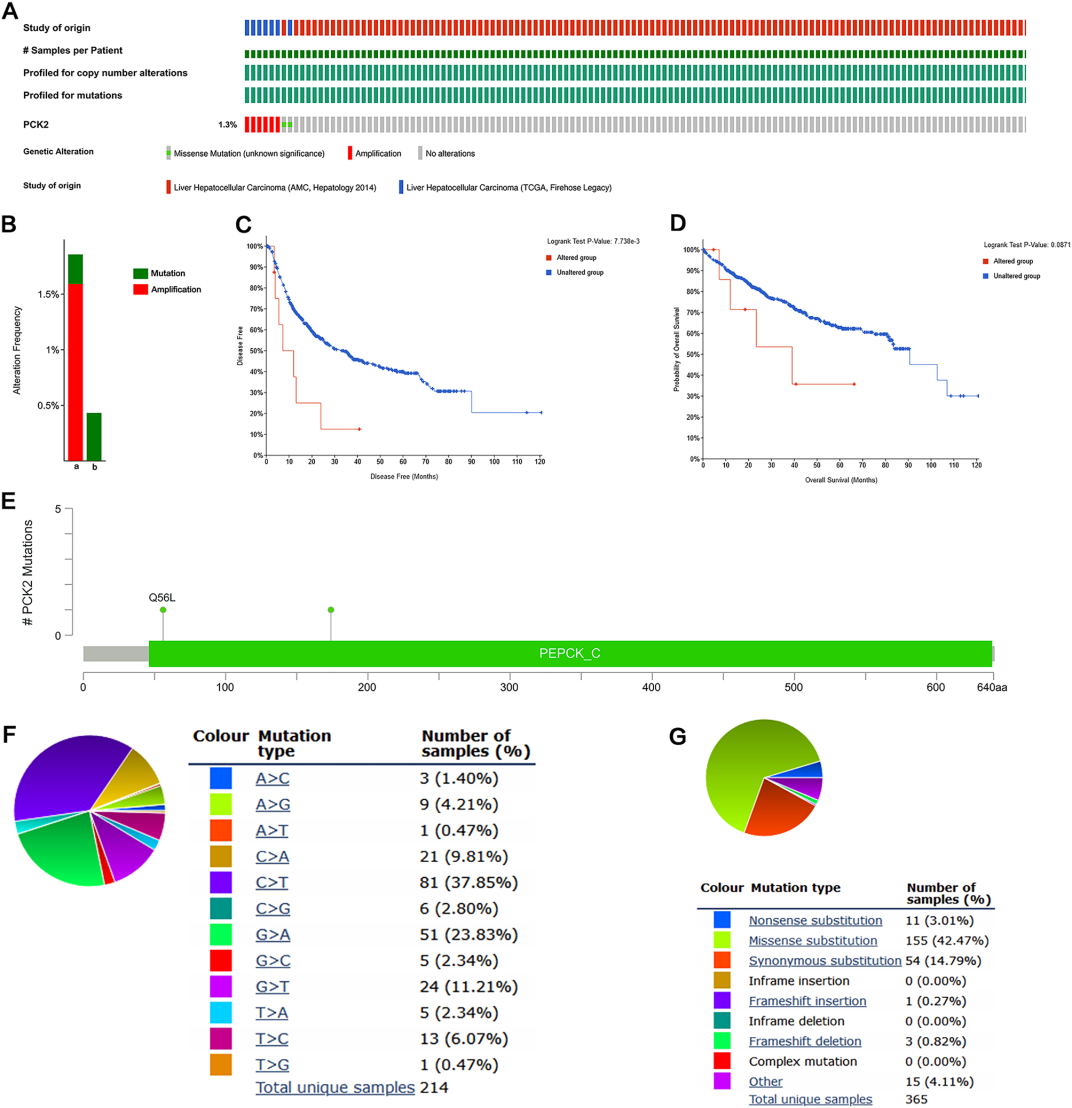
Genetic mutation in PCK2 in HCC. OncoPrint visual summary of alteration on a query of PCK2 (**A**). Summary of alterations in PCK2 in HCC from TCGA (**B**). Firehose Legacy (a); AMC Hepatology 2014 (b). Kaplan–Meier plots contrast overall survival and disease-free survival in patients with and without PCK2 genetic abnormalities (**C**,**D**). PCK2 mutations occur in HCC. Schematic illustration of PCK2 mutations in HCC (**E**). The mutation patterns in HCC in the COSMIC database (**F**,**G**).

The mutation frequency of PCK2 was evaluated in the cBioPortal database. A total of 608 HCC patients were evaluated from two data sets: TCGA, Firehose Legacy, and AMC Hepatology 2014. PCK2 gene alterations were found in 1.3 percent of HCC patients (Fig. 4A), with rates ranging from 0.43 percent (1/231) to 1.86 percent (7/377) (Fig. 4B). The frequency of such mutations is relatively low. Between patients with modifications and those without abnormalities, Cbioportal and log-rank tests revealed no significant differences in OS (P = 0.09) (Fig. 4D) and substantial differences in disease-free survival (P = 7.738e−3) (Fig. 4C). Further, the mutation types of PCK2 in HCC were assessed in the cBioPortal database, and samples from three databases were obtained, among which the mutations of PCK2 in HCC were primarily missense mutations (Fig. 4E). The types of mutations in PCK2 were evaluated in the COSMIC database. Figure 4 shows two mutation pie charts, in which about 42.47% of samples had missense replacement, 14.79% of samples had synonym replacement, and 3.01% of samples had nonsense replacement (Fig. 4G). Substitution mutations were most common in C > T (37.85 percent), followed by G > A (23.83%), G > T (11.21%) and C > A (9.81%) (Fig. 4F). In view of the low mutation rate and small sample size, PCK2 mutation in HCC patients may not be a factor leading to the carcinogenesis of low expression of PCK2.

### GO and KEGG pathway analyses

**Figure 5:**
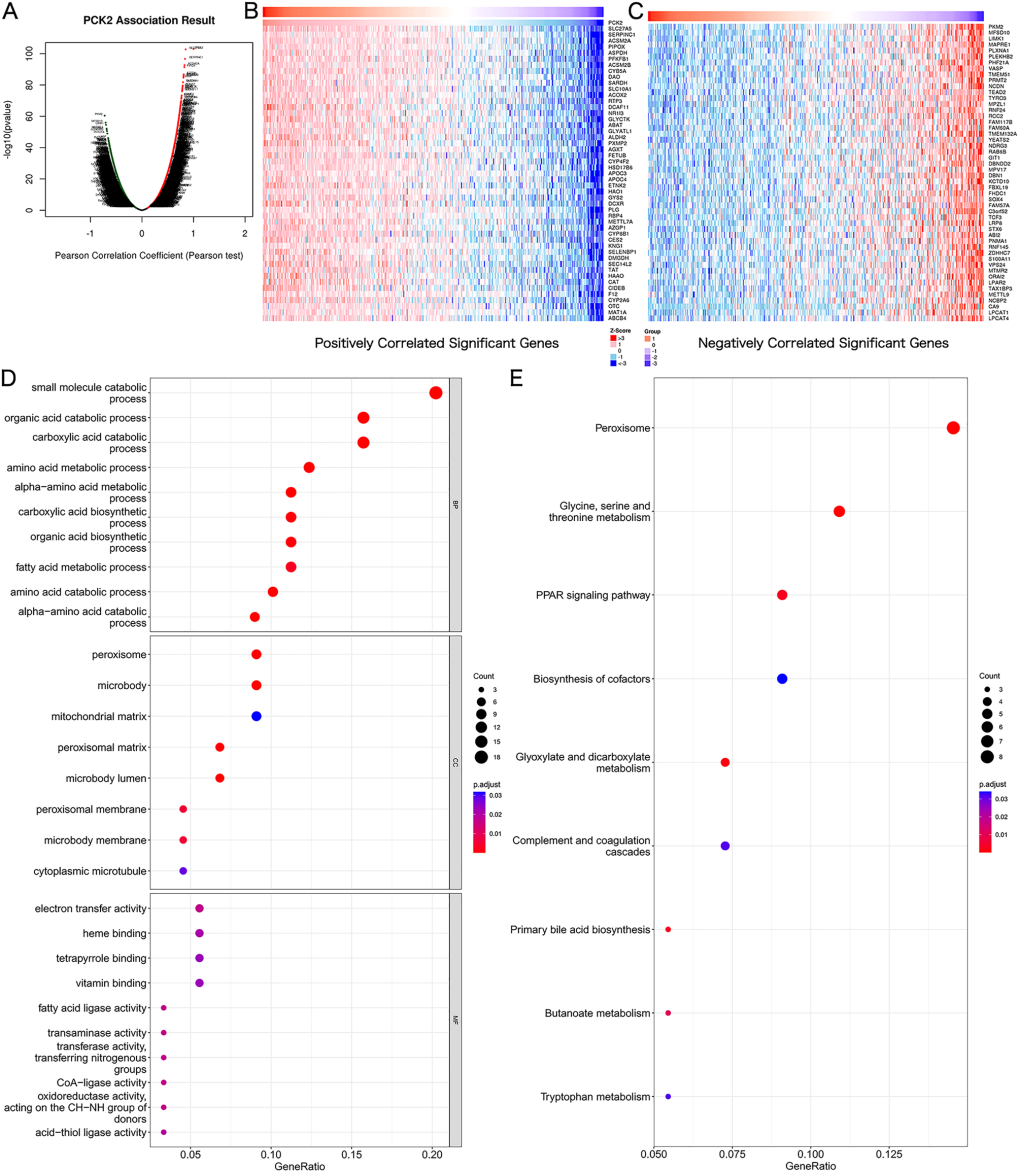
Functional enrichment analysis of PCK2 in HCC. The correlation analysis of PCK2 expression and its top 100 co-expressed gene network (**A**–**C**). GO and KEGG enrichment analysis of co-expressed genes(**D**,**E**).

In order to further understand the underlying mechanism of PCK2, we used LinkedOmics to obtain a significant correlation with the PCK2 gene (Fig. 5A). The heat map showed 50 gene sets with significant positive and negative associations with PCK2 (Fig. 5B,C), and further demonstrated the major functions of PCK2 with GO and KEGG. GO enrichment analysis includes three main functions, namely BP, CC, and MF, in which biological processes mainly include "small molecule catabolic process", "organic acid catabolic process" and "carboxylic acid catabolic process", cell components mainly include "peroxisome", "microbody" and "mitochondrial matrix", and molecular functions mainly include "electron transfer activity", "heme binding" and "tetrapyrrole binding" (Fig. 5D). The molecular pathway of KEGG mainly includes "Peroxisome", "Glycine, serine and threonine metabolism" and "PPAR signaling pathway" (Fig. 5E).

### GSEA identifies PCK2-related signaling pathways in HCC

**Figure 6:**
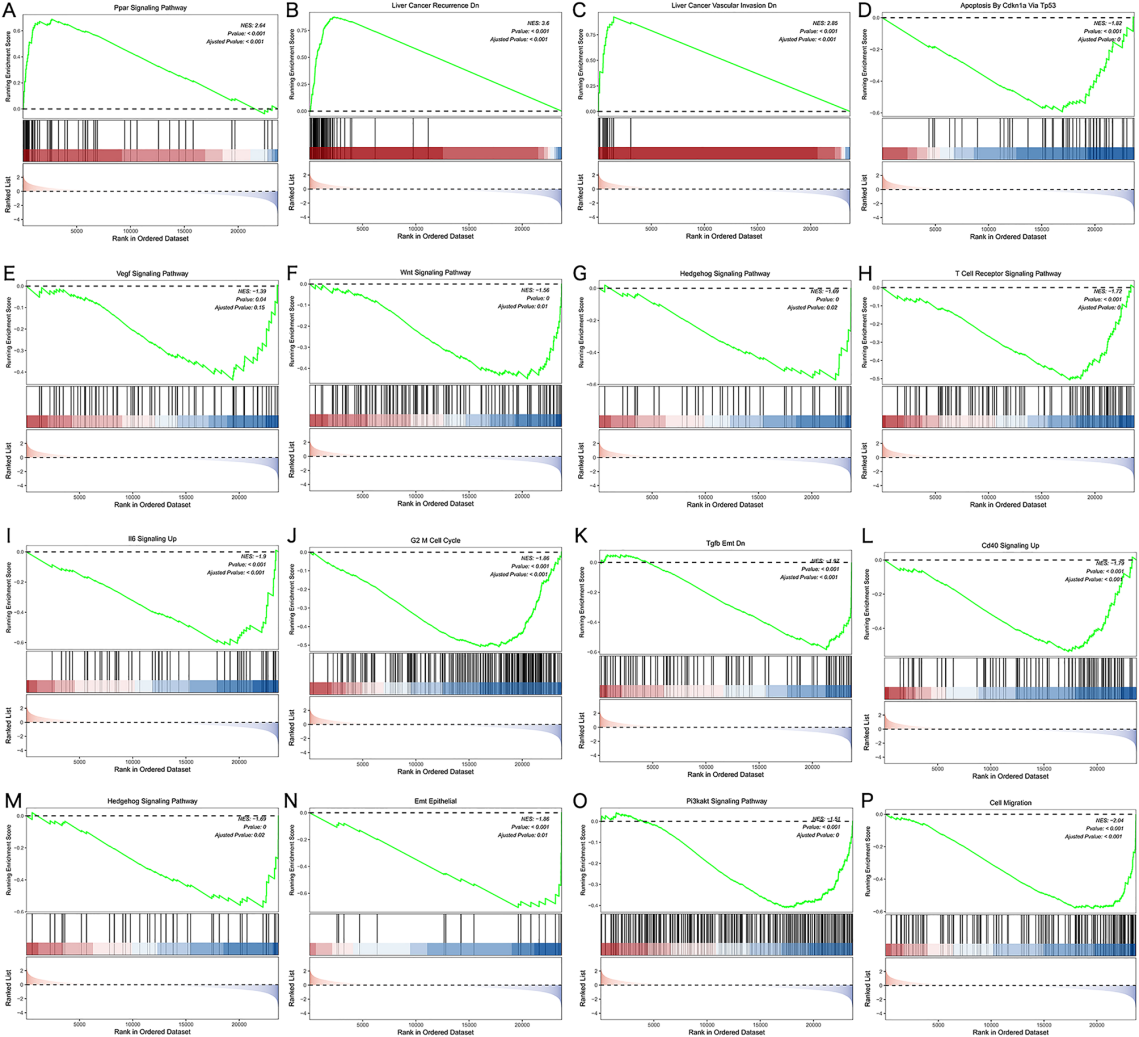
Identification of PCK2-related signaling pathways in HCC. Identification of PCK2-related signaling pathways by R software (**A**–**P**). *NES* normalized enrichment score, *NOM* nominal, *FDR* false discovery rate. Gene sets with NOM P-value < 0.05 and FDR q-value < 0.1 are considered as significant.

To investigate the potential molecular feature of PCK2 in HCC, GSEA was used to predict PCK2-related signaling pathways in samples with low and high expression levels. Among the 5334 pathways, 1300 were enriched in NOM P < 0.05 and FDR < 0.1, and NES >|1|. It is well known that many pathways are key members of liver regeneration, repair, metabolism, inflammation, and cancer, and have a certain influence on the proliferation, apoptosis, and metastasis of HCC cells. We know by sifting through the results, PCK2 mainly involves PPAR signaling pathway, recurrence, vascular invasion, apoptosis via tp53, VEGF signaling pathway, WNT signaling pathway, IL-6 signaling, G2 M cell cycle, TGF β EMT, CD40 signaling, Hedgehog signaling pathway, EMT epithelial, PI3K-AKT signaling pathway, and cell migration (Fig. 6).

### The expression of PCK2 was correlated with EMT characteristics in HCC

**Figure 7:**
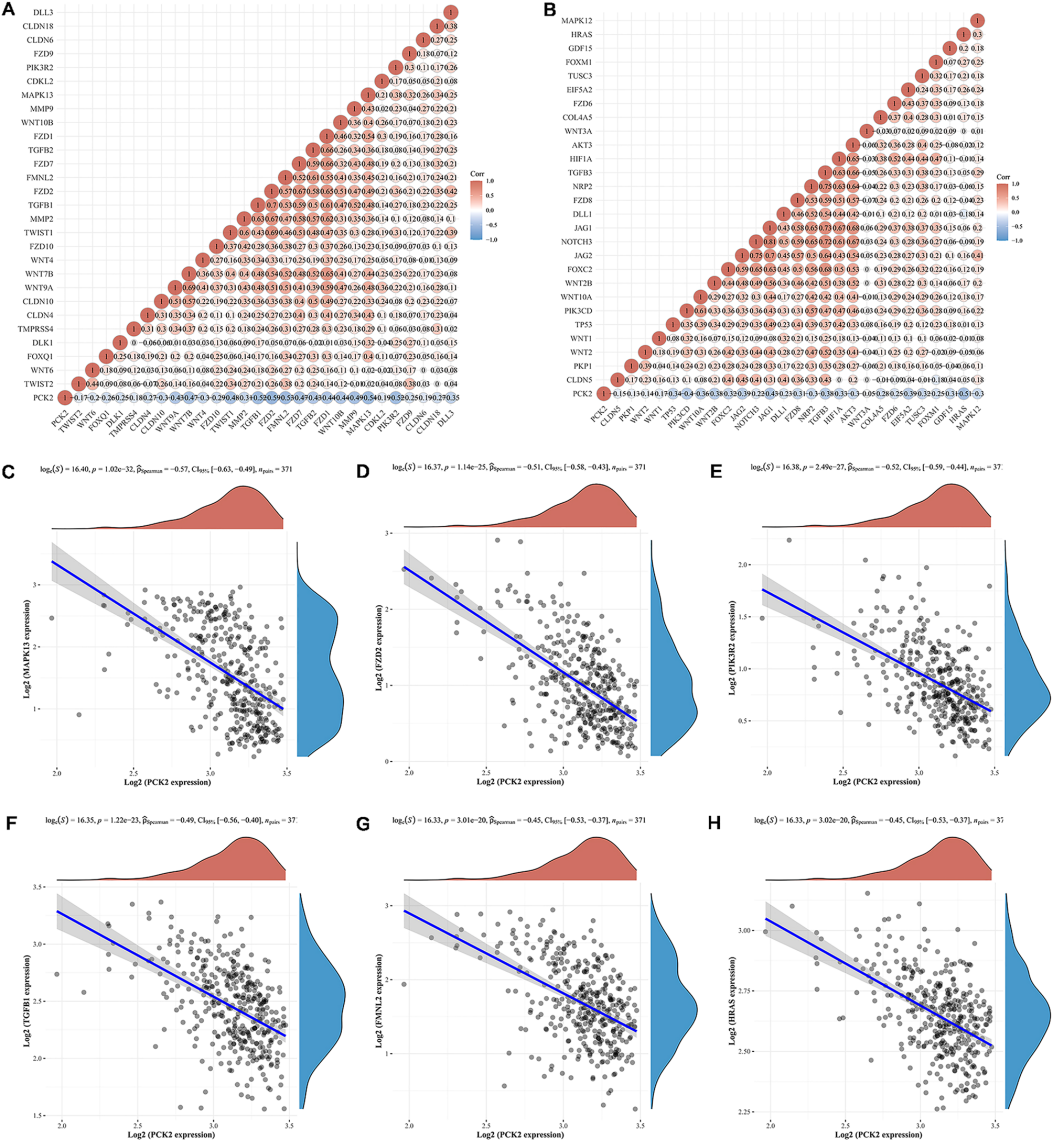
Relationship between PCK2 and EMT-related genes in GSEA pathway. The heat map shows the correlation between PCK2 and EMT pathway genes (**A**,**B**). Significant negative correlation between PCK2 and EMT-related genes in HCC (C-H).

PCK2 is connected with the Epithelial-mesenchymal transition (EMT) pathway (NOM P < 0.001, FDR < 0.1and NES >|1|), according to GSEA data (Fig. 6), and 55 genes of the EMT pathway have been obtained. The TCGA database was used to gather RNAseq data and clinical information for 371 HCC patients, and the pheatmap R software program was used to produce multi-gene correlation maps. The correlation between variables was described using Spearman's correlation analysis, with a P value of less than 0.05 regarded as statistically significant. PCK2 was found to be significantly negatively linked with EMT-related genes, according to the findings (Fig. 7A,B). R software package ggstatsplot was used to realize correlation analysis of the two genes. We found that the first six significantly related genes were HRAS, TGFB1, FMNL2, PIK3R2, FZD2, and MAPK13 (P < 0.001) (Fig. 7C–H).

### The association of PCK2 expression and immune infiltration in HCC

**Figure 8:**
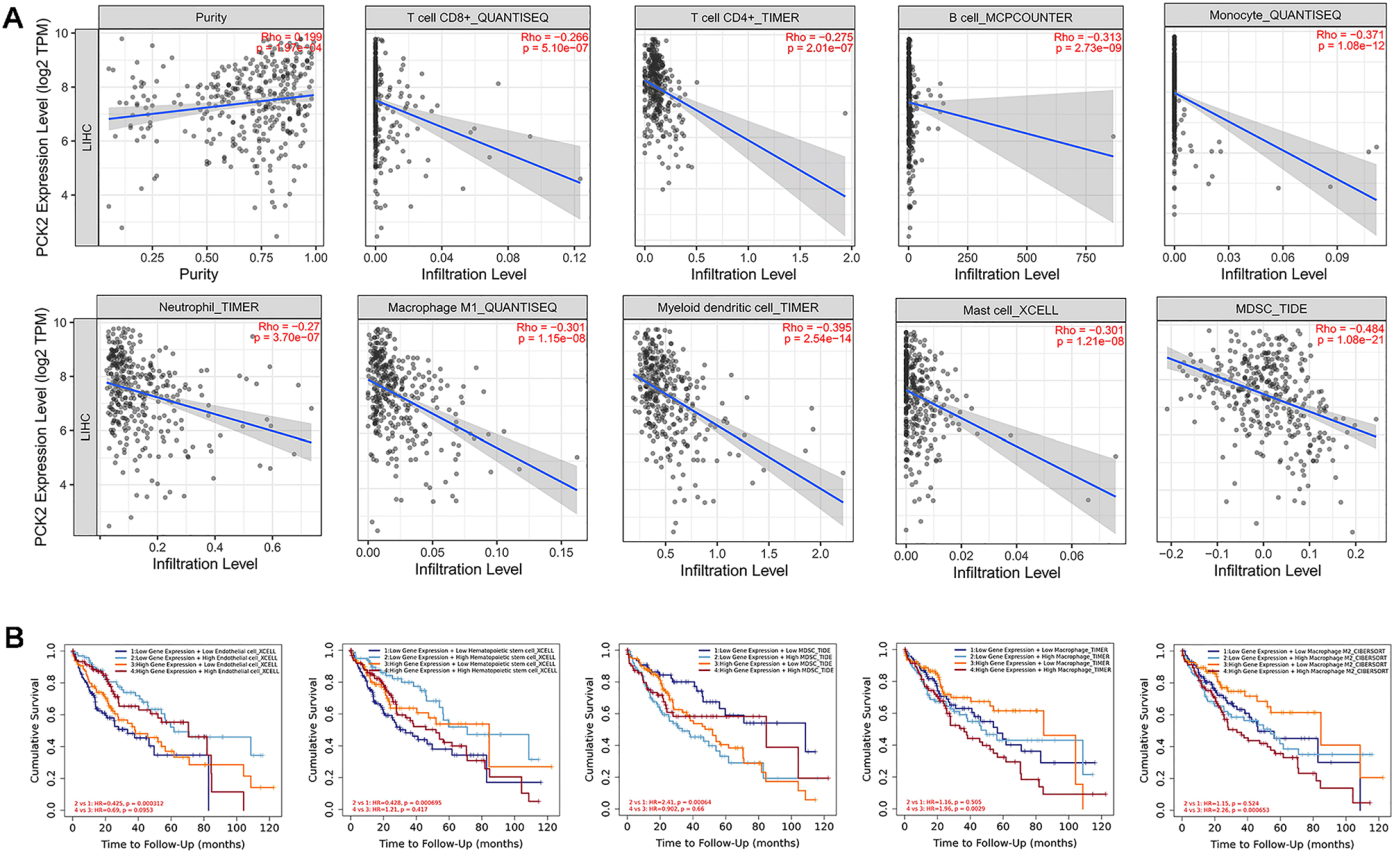
The linkage between PCK2 and immunological infiltration has been investigated for correlation and prognosis. The interaction between PCK2 expression and tumor immune infiltrating cells in HCC (**A**). The cumulative survival of PCK2 expression is related to the degree of immune infiltration in HCC (**B**).

Tumor-infiltrating lymphocytes have been shown in studies to be independent predictors of cancer sentinel lymph node status and prognosis, affecting survival in patients with a variety of cancers. In the TIMER database, the Spearman test was used to examine the relationship between PCK2 expression and immune infiltration. PCK2 expression was found to be related to tumor purity. Negative correlation was found with 9 kinds of infiltrating immune cells, including B cells (r = − 0.313, P = 2.73E−09), CD8 + T cells (R = − 0.266, P = 5.1E−07), CD4 + T cells (r = − 0.275, P = 2.01E−07) and macrophages M1(r = − 0.301, P = 1.15E−08), neutrophils (r = − 0.27, P = 3.70E−07), dendritic cells (P = − 0.395, P = 2.54E−14), monocytes (r = − 0.371, P = 1.08E−12), Mast cells (r = 0.301, P = 1.21E−08), MDSC (r = 0.484, P = 1.08E−21) (Fig. 8A). It has been shown above that PCK2 plays a certain role in the immune regulation of HCC.

Kaplan–Meier analysis of PCK2 expression level and the cumulative survival rate of different immune cell infiltration. We found that low expression of PCK2 in HCC led to increased infiltration of endothelial cells and hematopoietic stem cells, which greatly increased the overall survival of patients, while low expression of PCK2 in HCC led to increased infiltration of MDSC, which greatly reduced the overall survival of HCC patients. Further to that, we noted that high PCK2 expression resulted in reduced macrophage infiltration, particularly in M2 cells, which significantly improved patient overall survival, implying that PCK2 could become an immunotherapy target for M2 macrophages and improve the cumulative survival rate in HCC (Fig. 8B).

We also looked at the correction between PCK2 expression and immune cell gene markers (Table 2). Tumor purity was used to adjust correlation coefficients. PCK2 had a substantial negative connection with several immune cell gene markers. Among them, MRG1 (r = 0.448), the immune marker of M2 macrophages, attracted our attention, echoing the role of M2 in the survival analysis mentioned in the previous paragraph. All the results implied that PCK2 could not be overlooked in the immune infiltration during the progression of PCK2.

**Table 2:** Correlation analysis between PCK2 expression and immune cell markers in HCC.

Description	Gene markers	None		Purity	
		Cor	P	Cor	P
B cell	CD19	− 0.239	***	− 0.191	***
	CD27	− 0.284	***	− 0.212	***
	CD38	− 0.209	***	− 0.135	*
	CD79A	− 0.248	***	− 0.185	***
T cell (general)	CD3D	− 0.349	***	− 0.285	***
	CD3E	− 0.256	***	− 0.173	**
	CD2	− 0.261	***	− 0.18	***
CD8 + T cell	CD8A	− 0.179	***	− 0.093	0.085
	CD8B	− 0.229	***	− 0.155	**
Tfh	CXCR3	− 0.221	***	− 0.134	*
	CXCR5	− 0.272	***	− 0.225	***
	BCL6	− 0.07	0.177	− 0.092	0.089
	ICOS	− 0.292	***	− 0.223	***
Th1	IFN-γ (IFNG)	− 0.184	***	− 0.126	*
	IFN- (TNF)	− 0.263	***	− 0.205	***
	IL12RB2	0.065	0.212	0.091	0.091
	STAT4	− 0.167	**	− 0.116	*
	STAT1	− 0.148	**	− 0.124	*
	CD94 (KLRD1)	0.050	0.340	0.125	*
	BET (TBX21)	− 0.047	0.369	0.038	0.484
Th2	STAT6	0.112	*	0.094	0.082
	CCR3	− 0.341	***	− 0.312	***
	CD4	− 0.052	0.315	0.031	0.571
	STAT5A	− 0.234	***	− 0.194	***
Th9	TGFBR2	0.052	0.315	0.064	0.239
	IRF4	− 0.326	***	− 0.281	***
	SPI1	− 0.352	***	− 0.315	***
Th17	CD161 (KLRB1)	− 0.177	***	− 0.086	0.112
	CD121A(IL1R1)	− 0.041	0.435	− 0.03	0.573
	STAT3	− 0.121	*	− 0.108	*
Th22	CCR10	− 0.26	***	− 0.236	***
	AHR	0.007	0.889	0.006	0.910
Treg	CD25 (IL2RA)	− 0.336	***	− 0.301	***
	CCR8	− 0.178	***	− 0.131	*
	FOXP3	0.174	***	0.215	***
	CD127 (IL7R)	− 0.125	*	− 0.057	0.288
Exhausted T cell	PD-1 (PDCD1)	− 0.373	***	− 0.335	***
	Tim-3 (HAVCR2)	− 0.352	***	− 0.315	***
	CTLA4	− 0.392	***	− 0.339	***
	LAG3	− 0.176	***	− 0.123	*
	GZMB	− 0.134	**	− 0.08	0.139
M1 Macrophage	INOS (NOS2)	0.149	**	0.159	**
	IRF5	− 0.109	*	− 0.134	*
	COX2 (PTGS2)	− 0.231	***	− 0.169	**
M2 Macrophage	ARG1	0.433	***	0.448	***
	VSIG4	− 0.111	*	− 0.04	0.463
	MS4A4A	− 0.12	*	− 0.045	0.407
TAM	CD80	− 0.315	***	− 0.284	***
	PDCD1LG2	− 0.008	0.883	0.091	0.091
	CD40	0.183	***	0.215	***
	TLR7	− 0.27	***	− 0.22	***
	CCL2	− 0.159	**	− 0.061	0.256
	IL10	− 0.249	***	− 0.196	***
Monocyte	CD86	− 0.316	***	− 0.261	***
	CD115 (CSF1R)	− 0.278	***	− 0.229	***
NK cell	NCAM1	− 0.162	**	− 0.119	*
	KIR2DL1	0.005	0.920	0.023	0.667
	KIR2DL3	− 0.049	0.346	− 0.021	0.695
	KIR2DL4	− 0.132	*	− 0.096	*
	KIR3DL1	0.070	0.178	0.095	*
	CD7	− 0.328	***	− 0.287	***
	XCL1	− 0.202	***	− 0.177	***
Neutrophil	CD66b (CEACAM8)	− 0.13	*	− 0.126	*
	CD11b (ITGAM)	− 0.245	***	− 0.215	***
	CD15 (FUT4)	− 0.36	***	− 0.337	***
	CCR7	− 0.154	**	− 0.07	0.196
	MPO	− 0.153	**	− 0.072	0.180
Dendritic cell	CD1C	− 0.164	**	− 0.093	*
	CD141(THBD)	− 0.049	0.343	0.035	0.519
	HLA-DPB1	− 0.25	***	− 0.187	***
	HLA-DQB1	− 0.219	***	− 0.15	**
	HLA-DRA	− 0.212	***	− 0.148	**
	HLA-DPA1	− 0.203	***	− 0.138	*
	BDCA-4 (NRP1)	− 0.264	***	− 0.241	***
	CD11c (ITGAX)	− 0.33	***	− 0.295	***

*P < 0.05; **P < 0.01; ***P < 0.001.

### Correlation between PCK2 expression and immune checkpoints in HCC

**Figure 9:**
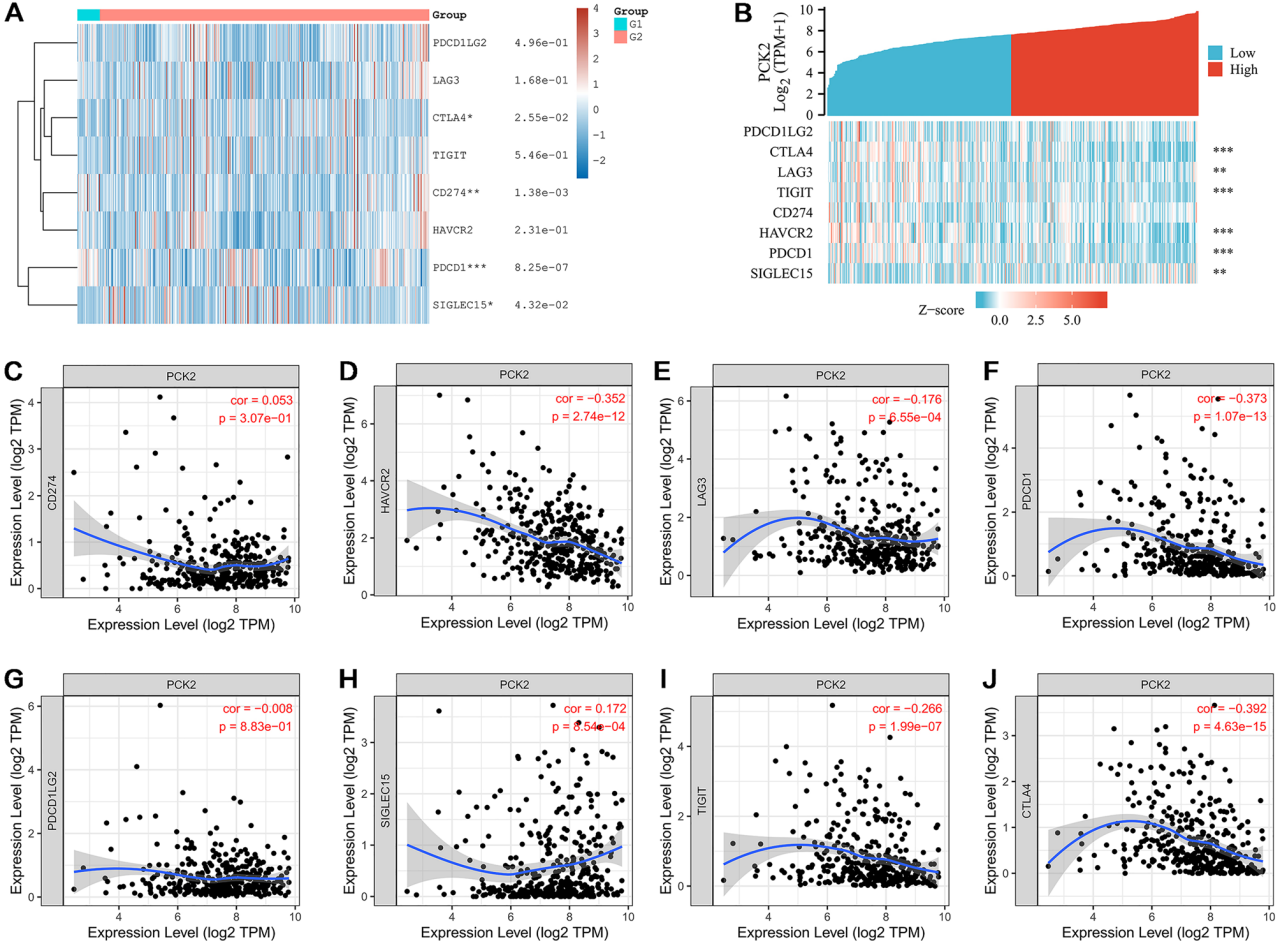
PCK2-related immune checkpoint gene expression in hepatocellular carcinoma. Heat maps of PCK2 immune checkpoint-related gene expression were presented in the GSE112790 and GSE102079 datasets (**A**). TCGA database showed the correlation between PCK2 and each immune checkpoint (**B**). The association between PCK2 and 8 immune checkpoints was evaluated in the TIMER database (**C**–**J**). *P < 0.05; **P < 0.01; ***P < 0.001.

Based on the GSE112790 and GSE102079 data sets of HCC screening, the expression heatmaps of immune checkpoint genes in normal liver tissue and hepatocellular carcinoma are displayed, in which various colors represented the expression characteristics in different samples (Fig. 9A). Further in the TCGA database, the expression of immune checkpoint genes in the two groups was visualized according to the high and low expression of PCK2 in HCC samples (Fig. 9B). Regarding the potential carcinogenic role of low PCK2 expression in HCC, the relationship between PCK2 and the immune checkpoint was further assessed in the TIMER database. According to the above results, PCK2 has the greatest correlation with PD-1, CTLA-4, HAVCR2, and TIGIT (Fig. 9C–J).

### Knockdown of PCK2 promoted the proliferation, migration and invasion of HCC cells

**Figure 10:**
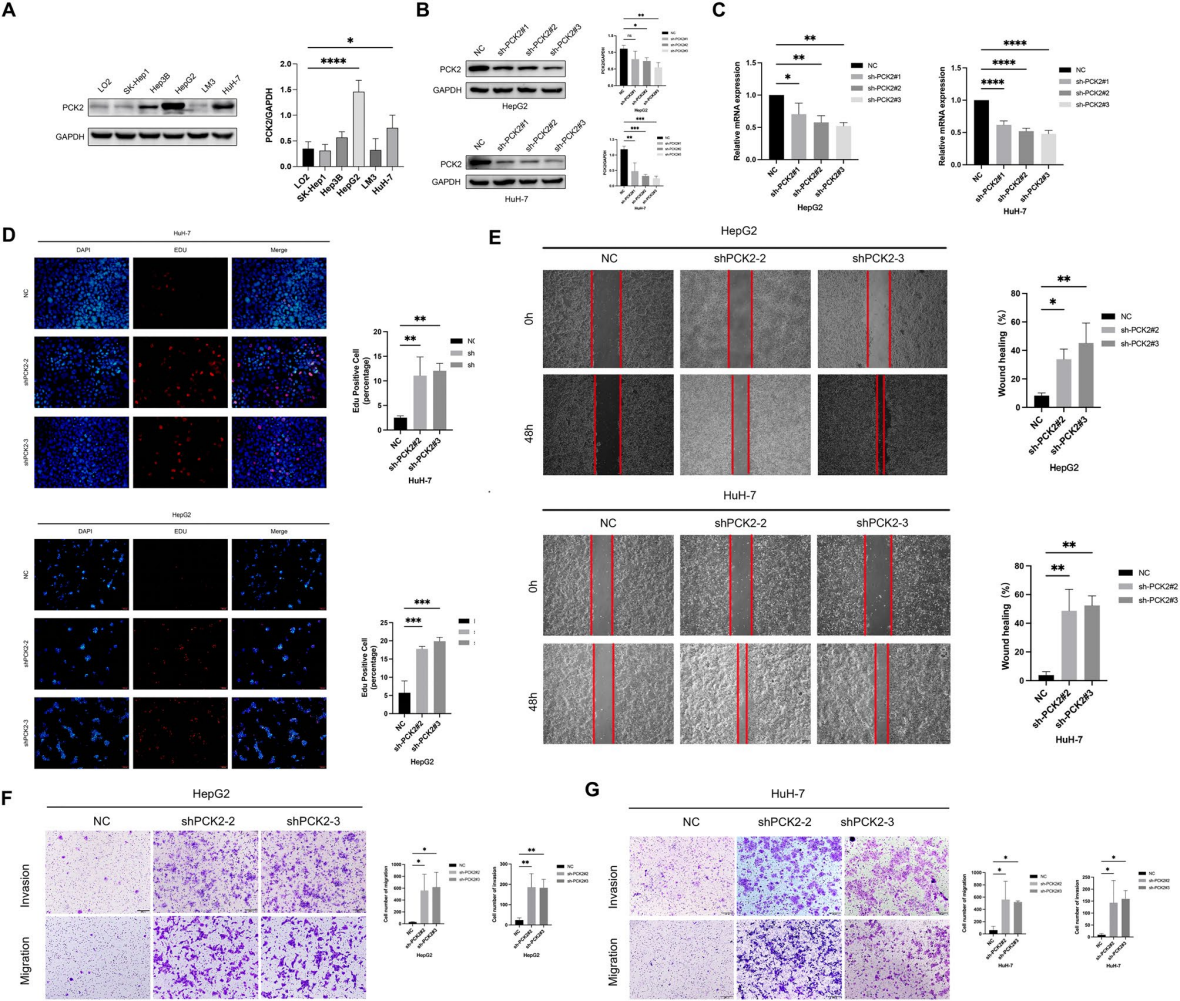
PCK2 regulates the proliferation and migration of HCC cells. Expression of PCK2 in different hepatoma cell lines (**A**). The knockdown effect of PCK2shRNAs in HepG2 cells and HuH-7 cells was detected by qPCR and western blot (**B**,**C**). The proliferation function of PCK2 in HepG2 cells and HuH-7 cells was verified by EDU assay (**D**). Wound-healing assays and transwell assays examined the effect of PCK2 knockdown on cell migration (**E**–**G**). *NC* negative control, *shRNA* PCK2 shRNA; ***P < 0.001.

**Figure 11:**
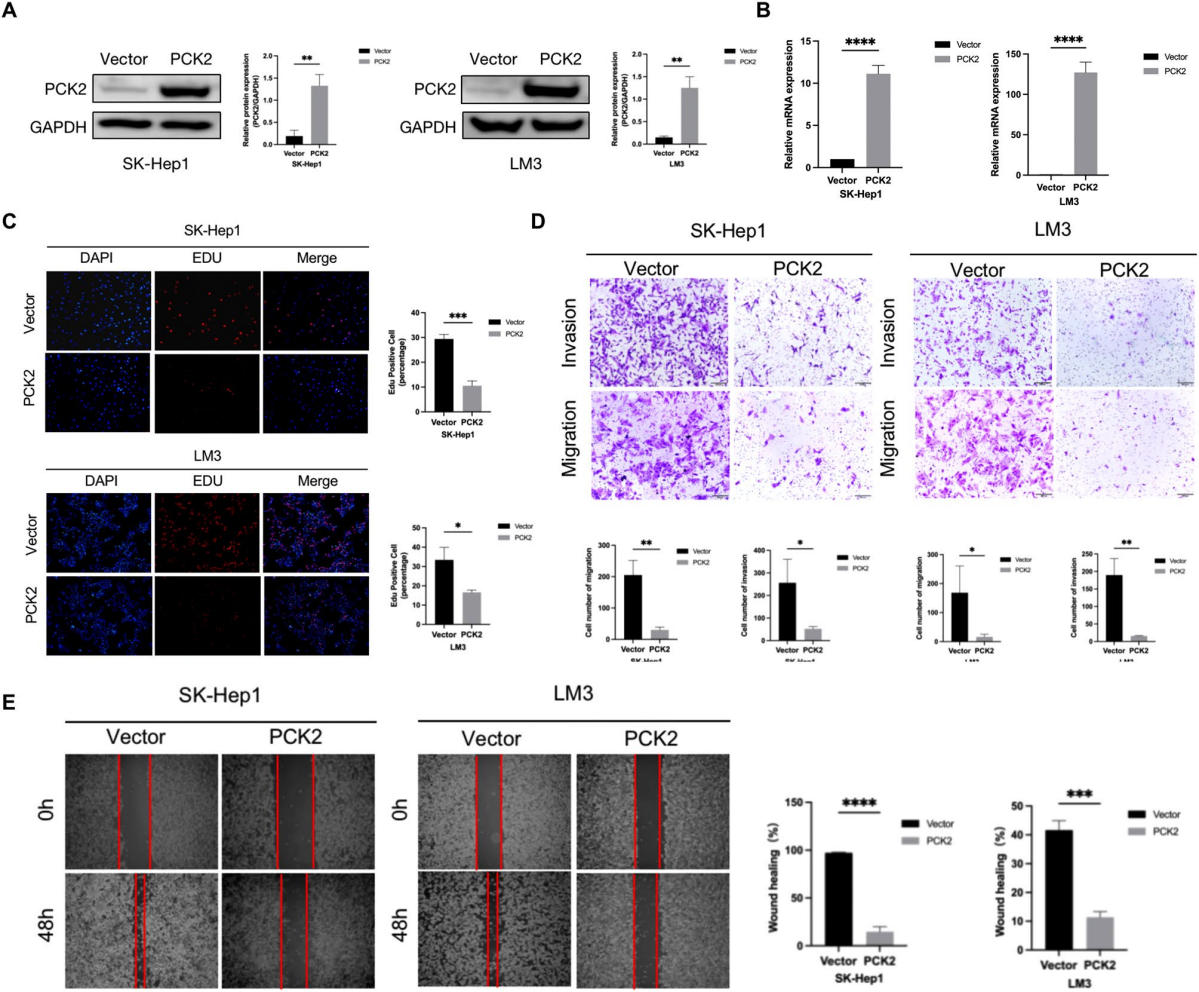
PCK2 regulates the proliferation and migration of HCC cells. The effect of overexpression of PCK2 on SK-Hep1 and LM3 cells was detected by qPCR and western blot (**A**,**B**). The proliferation function of PCK2 in SK-Hep1 and LM3 cells was verified by EDU assay (**C**). The effect of PCK2 overexpression on cell invasion and migration was detected by wound healing assay and transwell assay (**D,E**). *Vector blank control*, *PCK2 overexpression of PCK2 gene*; ***P < 0.001.

Previous studies have shown that PCK2 is low expressed in HCC, and patients with low expression of PCK2 have a poor survival prognosis. PCK2 plays an important role in HCC growth, migration and invasion, EMT, apoptosis and participation in various pathways. Therefore, in order to further explore the biological function of PCK2 in HCC progression, we conducted cell line screening and found that PCK2 was relatively highly expressed in HepG2 and HuH-7 cells (Fig. 10A). We knocked PCK2 expression at mRNA and protein levels in HepG2 and HuH-7 cells and obtained the two PCK2 shRNAs with the best knockdown effect (Fig. 10B,C). We then performed an EDU assay to assess cell proliferation. The results showed that the proliferation rate of HepG2 and HuH-7 cells increased after PCK2 was knocked down (Fig. 10D). In addition, as demonstrated by the wound-healing assays and transwell assays, PCK2 knockdown also promotes the ability of liver cancer cells to migrate and invade (Fig. 10E–G). All in all, PCK2 knockdown promoted the biological functions of liver cancer cell proliferation,invasion and migration. Further, we overexpressed PCK2 expression in SK-Hep1 and LM3 cells and verified up-regulated expression of PCK2 mRNA and protein levels in both cell lines (Fig. 11A,B). We then performed EDU, wound healing, and transwell tests. Experiments showed that overexpression of PCK2 could inhibit the proliferation, migration and invasion ability of liver cancer cells (Fig. 11C–E). In conclusion, overexpression of PCK2 inhibits the biological functions of hepatoma cell proliferation, invasion and migration.

**Figure 12:**
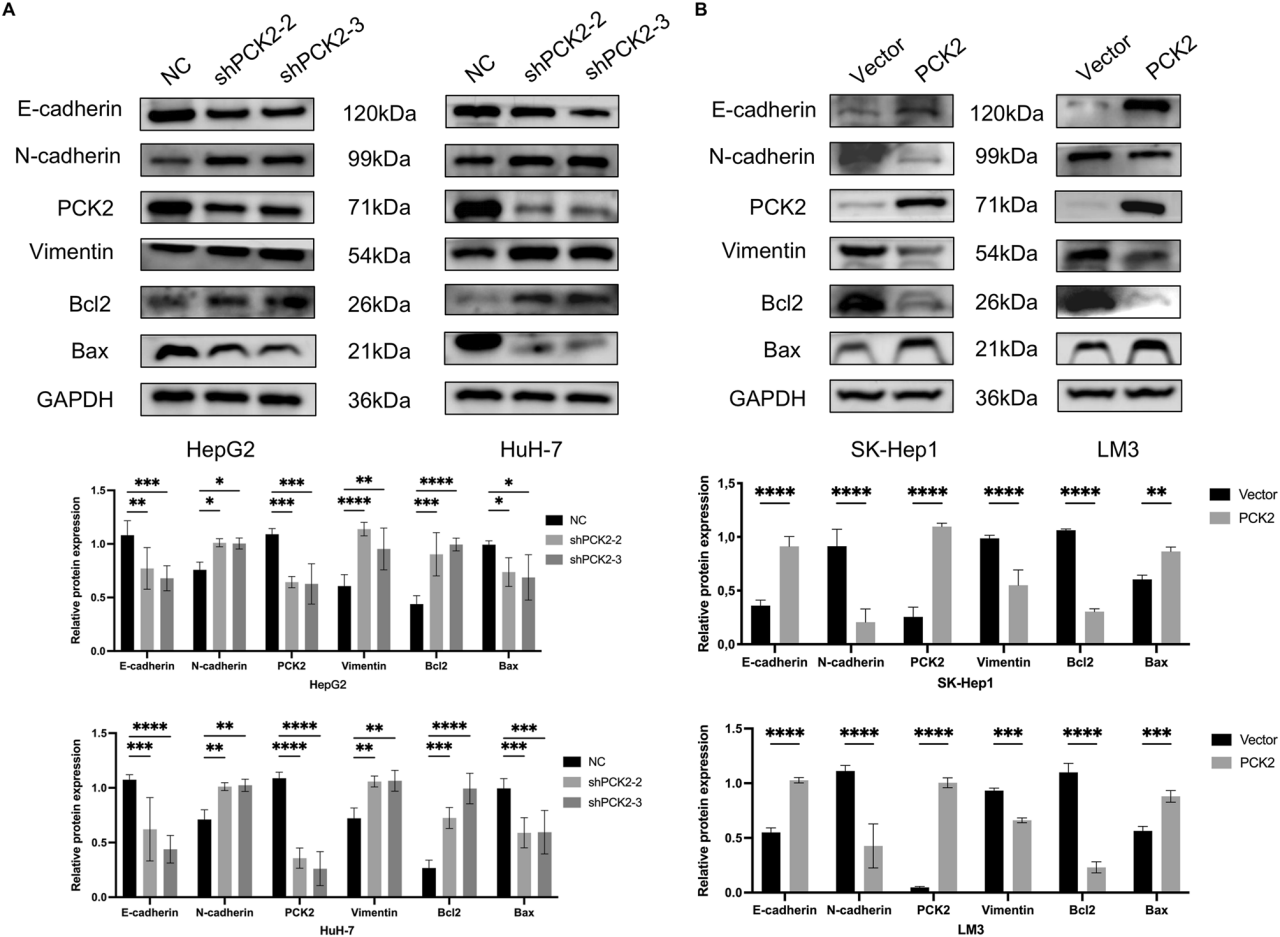
Effects of knockdown and overexpression of PCK2 on E-cadherin, N-cadherin, vimentin and apoptosis-related proteins. Expression changes of EMT and apoptosis protein after PCK2 knockdown (**A**). Changes of protein expression after overexpression of PCK2 (**B**).

In order to further verify the correlation between PCK2 protein and EMT and apoptosis, we verified the relationship between PCK2 and EMT-related proteins E-cadherin, N-cadherin and vimentin as well as apoptosis-related proteins Bcl2 and Bax by western blot. We found that knockdown of PCK2 significantly increased the levels of N-cadherin, vimentin and Bcl2 proteins, and decreased the levels of e-cadherin and Bax proteins (Fig. 12A,B).

## Discussion

At present, Hepatocellular carcinoma is still a global and international health issue with elevated concern. Despite significant advancements in systemic therapy, such as molecular-targeted medicines, it continues to have one of the worst prognoses due to drug resistance and frequent recurrence and metastasis. Hence, recognizing the pathogenesis and molecular pathways of HCC development is critical for developing novel therapeutic interventions. In recent years, there is growing evidence that changes in metabolic pathways are closely linked to cancer, and many metabolism-related genes/proteins^15,16^, such as the PCK2 gene, perform non-metabolic functions. At the same time, the liver is an immunological organ and the study of the immune microenvironment and immune checkpoint on liver cancer has gradually entered the public's attention^17^. Therefore, exploring the expression, survival prognosis, mutation, functional enrichment, metastasis, immune invasion, immune checkpoint, and proliferation migration and invasion of PCK2 in liver cancer will lay a foundation for further understanding of its mechanism.

PCK2 promotes cancer growth in vivo by mediating tumor cell glucose restriction adaption^18^. PCK2 expression is elevated in a variety of human tumor types, including lung, breast, and prostate cancer^19–24^, but the molecular mechanism of PCK2 in HCC is comparatively sporadic. How is the expression of PCK2 in HCC, whether it is mutated, and whether it is related to survival prognosis? What role does it play in tumorigenesis, development, and metastasis, how does it relate to immune invasion, and whether it can serve as an immune checkpoint? Through the database, we demonstrated that PCK2 is underexpressed in HCC, and the percentage of PCK2 gene changes is 1.3%, which is dominated by missense mutations. Gene mutations are closely associated with tumors and are often associated with poor tumor prognosis, but the proportion of PCK2 gene changes in HCC is too low, and gene changes are not significantly associated with poor OS, and are statistically significant with disease-free survival. However, due to the low variation rate of PCK2 and the large difference between the number of people with and without variation, the reliability of K-M analysis results is poor, and the variation of PCK2 in HCC patients may not be a factor leading to the carcinogenesis of low expression of PCK2. It has been suggested that PCK2 gene mutation causes primary angle-closure glaucoma (PACG) by disrupting the AKT/GSK3α signaling pathway^25^. Loss of the p53-Mir-200C-PCK2 axis may provide metabolic benefits that provide a cornerstone for cancer cell growth, leading to the growth of basal-like breast cancer (BLBC)^26^. Though, the research on PCK2 mutation in HCC is still very few, so we need to explore it at a time.

Since the TCA cycle is the main means by which cancer cells meet their balance requirements for bioenergy, biosynthesis, and redox, metabolic reprogramming has become increasingly important in recent years for promoting tumor survival and proliferation. Numerous cancers have been related to TCA cycle rewiring, and PCK2 is an essential modulator of TCA cycle flux that promotes cell proliferation both in vivo and in vitro by boosting the use of anabolic glucose and glutamine^8^. We have explored the potential of the TCA cycle as a metabolic target for HCC treatment^27^. Studies have also demonstrated that glucose metabolism gene expression can be exploited as a possible prognostic marker and/or therapeutic target, which could aid in the development of more specific treatments for HCC patients^28^. HCC is characterized by changes in metabolic pathways^29^, with major modifications in the TCA cycle and related enzymes linked to cancer cell alteration and development, and maintaining the redox condition essential for tumor survival and proliferation. PCK2 is involved in metabolism, particularly in the TCA cycle, which opens up new avenues for the HCC treatment^30^.

The findings of GSEA could lead to fresh insights into the mechanism by which PCK2 alters many biological activities. As a consequence of the GSEA results in our work, several critical signaling pathways were active, including EMT, Hedgehog Signaling pathway, Wnt signaling pathway, PI3K-AKT signaling pathway, MAPK signaling pathway, Notch pathway, and AKAP13 pathway, were activated in the low PCK2 expression group. EMT is a prodromal phase that cancer cells use to escape the main tumor, infiltrate neighboring tissues, and spread to distant locations^31^. EMT is critical in the early stages of cancer growth when there is no cell-to-cell contact as a result of E-cadherin ablation and gain enhanced mobility to disseminate into adjacent or distant groups^32^. Because strong inducers of EMT can influence fibrosis and carcinogenesis, epithelial plasticity is already a hot topic in HCC, including elevated cytokine levels in advanced HCC^33–37^. ROS-mediated atypical hedgehog signaling induced by hypoxia may play an essential role in EMT, providing a promising marker for the prevention and therapy of liver cancer. Hypoxia accelerates the invasiveness of HCC cells, involving oxidative stress, EMT, and atypical Hedgehog signal transduction^38^. The Sonic hedgehog and Wnt pathways are both activated in a network model of TGF signaling in epithelial-mesenchymal transformation in hepatocellular carcinoma^39^. The activation mechanism, signal transduction molecules, and impact on cancer genes, as well as key proteins that control cell proliferation, all have a substantial impact on PI3K/AKT-mediated signal transduction^40,41^. The mitogen-activated protein kinase (MAPK) is involved in a wide range of biological processes, including autophagy and apoptosis in HCC^42^. Facilitating Notch-related EMT in vivo altered the tumor immune microenvironment^43^. When activated by different ligands, Notch signaling performs a pleomorphic role in HCC, affecting tumor growth, invasion ability, and stem-cell-like characteristics, depending on cell type^44^. APK13 expression in HCC could be made as a biomarker for liver cancer diagnosis and prognosis^45^. With advancements in tumor biology and molecular genetic analyses, a growing number of signaling pathways and molecular mechanisms have been discovered as causes of HCC start and progression, and have thus become prospective targets for pharmaceutical research prediction^46^. As our results suggest, PCK2 knockdown can promote EMT pathway, and E-cadherin expression decreases and N-cadherin and Vimentin expression increases after PCK2 knockdown. The reverse was observed after overexpression of PCK2. The decreased expression of PCK2 may promote EMT by activating cytokines, a series of signaling pathways and altering immune infiltration, and ultimately lead to tumor immune evasion, low survival rate and increased invasivity in patients with liver cancer.

Liver cancer may benefit from immunotherapy, and immune-based therapies such as immune checkpoint inhibitors have changed the course of systemic cancer treatment^47^. Yet, little is known about PCK2 in liver cancer and immune invasion and checkpoint. The aforementioned study was carried out to look into the interaction between PCK2 and the immune microenvironment, and the results revealed that PCK2 was linked to distinct immune infiltration and immunological checkpoints in HCC. We demonstrated a significant correlation between PCK2 and macrophage M2, monocyte, and MDSC in both databases. The recruitment of MDSCs in HCC has been reported before^48^, but whether it is related to PCK2 is unknown. A new study also indicates that PCK2 exerts a non-metabolic component in regulating LPS-induced inflammation and sheds light on the regulatory mechanisms that govern inflammatory responses in Kupffer cells, which are intimately associated with liver cancer^49^. Since a rising proportion is being recognized as advanced, immune checkpoint inhibitors may allow patients to live longer. The involvement of immunological checkpoint proteins such as programmed cell death protein-1 (PD-1) and cytotoxic T lymphocyte-associated antigen 4 (CTLA-4) in liver cancer is becoming increasingly well-known^50,51^. TIGIT is a CD8 + effector memory T cell inhibitory molecule that interacts with PVR to inhibit anti-tumor immune responses, and PVRL1/TIGIT inhibitors and anti-PD1 antibodies could be developed to treat HCC^52^. TIGIT is a novel suppressive immune checkpoint that is essential in adaptive immune-mediated tumor progression and liver tolerance to infection and tumor cell invasion^53^. TIGIT/PD1 co-blocking increased the function of CD8 + TILs in vitro, but these CD8 + TILs were ineffective against PD1 blocking alone. As a result, inhibiting TIGIT/PD1 together could be a potential immunotherapy treatment for HCC patients^54^. PDCD1LG2 may be a prognostic biomarker of HCC and may be associated with tumor immune escape^55,56^. The role of LAG-3 in inhibiting HBV-specific cell-mediated immunity in HCC could pave the way for new cancer treatments^57^. More and more studies have been conducted on HAVCR2 as a target for liver cancer immunotherapy^58–64^. Glucose and PEPCK have been shown to modulate NFAT and C-myc activity via the PEP/Ca2 + axis, which is critical for immune activation in tumors during nutrient competition^65^. Hence, PCK2 is likely to act as an auxiliary immunological checkpoint and regulate immune infiltration, providing a unique strategy for HCC immunotherapy.

In conclusion, our study found low expression of PCK2 in HCC through bioinformatics, and found that low expression of PCK2 is an indicator of poor prognosis. The possible functions and pathways of PCK2 were explored by GO, KEGG and GSEA functional enrichment analysis. Next, we conducted cell experiments to verify the possible biological functions of PCK2, such as proliferation, invasion and migration, and verified its correlation with EMT protein and apoptosis protein by Western blot. At the same time, we also understood the relationship between PCK2 and variation, immune infiltration and immune checkpoint, and explored the occurrence and development of PCK2 in HCC from multiple perspectives. Therefore, to study the effects of PCK2 on malignant behaviors such as liver cancer proliferation, invasion and metastasis, and find that PCK2 affects the changes of EMT and other related signaling pathways, which will help us better understand the role of PCK2 in the invasion and metastasis of liver cancer, and provide new ideas for the treatment strategy of liver cancer metastasis. Our study is only a small step in the study of PCK2 in HCC. In order to clarify the possible role of PCK2 in HCC development, more studies are needed in the future to elucidate the molecular mechanism of PCK2 in HCC.

## Conclusion

To summarize, we know that the down-regulation of PCK2 expression in HCC predicts poor prognosis and is related to the immune invasion. Targeting PCK2 for targeted or combination immunotherapy could be an efficient strategy. PCK2 and its related genes are mainly enriched in the growth, invasion, metastasis, EMT, PPAR, WNT, PI3K/AKT and other biological signaling pathways of liver cancer cells, which opens a new window for the treatment strategy of liver cancer. After all, our grasp of genomic changes in PCK2 start and progression is rudimentary, and the molecular genetic modifications unique to HCC require further consideration summary, the aberrant expression of certain genes is crucial in the formation of hepatocellular carcinoma, which involves the determination of the most appropriate treatment and timing of various therapies at the level of molecular mechanism to obtain the best therapeutic effect. PCK2, which is currently poorly studied, can one day become a novel and significant prognostic biomarker and therapeutic target.

### Supplementary Information

Supplementary Information 1.

Supplementary Information 2.

Supplementary Information 3.

Supplementary Information 4.

Supplementary Information 5.

Supplementary Information 6.

Supplementary Information 7.

Supplementary Information 8.

Supplementary Information 9.

Supplementary Information 10.

Supplementary Information 11.

Supplementary Information 12.

## Data Availability

The datasets generated during and analyzed during the current study are available in the TCGA, GEO and ICGC repository, https://genomecancer.ucsc.edu/, www.ncbi.nlm.nih.gov/geo.https://dcc.icgc.org/.

## Abbreviations

PCK2/PEPCK-M: Phosphoenolpyruvate carboxykinase 2, mitochondrial
PCK1/PCPEK-C: Phosphoenolpyruvate carboxykinase 1, cytoplasm
PEPCK: Phosphoenolpyruvate carboxykinase
HCC: Hepatocellular carcinoma
TCGA: The Cancer Genome Atlas
GEO: Gene Expression Omnibus
COSMIC: Cancer somatic mutation catalog database
GSEA: Gene set enrichment analysis
CPTAC: Clinical proteomic tumor analysis consortium
TIMER: Tumor immune estimation resource
GO: Gene ontology
KEGG: Kyoto encyclopedia of genes and genomes
GSEA: Gene set enrichment analysis
HPA: Human Protein Atlas
ANOVA: Analysis of variance
EMT: Epithelial–mesenchymal transition
CSC: Cancer stem cell
TCA: Tricarboxylic acid cycle
PEP: Phosphoenolpyruvate
OS: Overall survival
DSS: Disease-specific survival
DFS: Disease-free survival
PFS: Progression-free survival
HRs: Hazard ratios
CIs: Confidence intervals
COAD: Colon adenocarcinoma
LIHC: Liver hepatocellular carcinoma
KIRP: Kidney renal papillary cell carcinoma
KICH: Kidney chromophobe
LUAD: Lung adenocarcinoma
CHOL: Breast invasive carcinoma
KIRC: Kidney renal clear cell carcinoma
PACG: Primary angle-closure glaucoma
BLBC: Basal-like breast cancer
NES: Normalized enrichment score
NOM: Nominal
FDR: False discovery rate
