# Supplementary figures and images for: Association of mitochondrial phosphoenolpyruvate carboxykinase with prognosis and immune regulation in hepatocellular carcinoma
Source: Sci Rep. 2024 Jun 18;14:14051. doi: 10.1038/s41598-024-64907-7 (PMC11189538; doi:10.1038/s41598-024-64907-7)

## Complementary immunohistochemistry

1. P1

N1

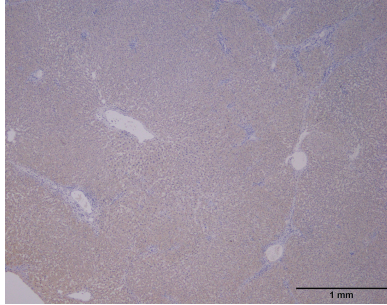

4X

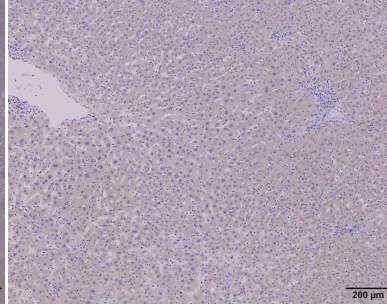

10X

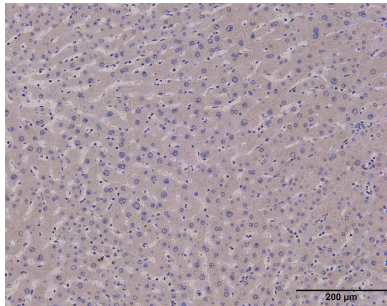

20X

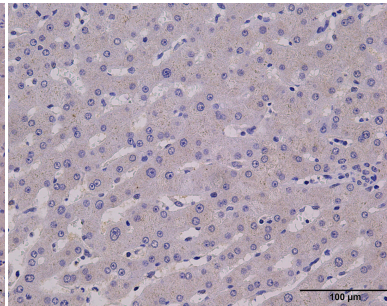

40X

T1

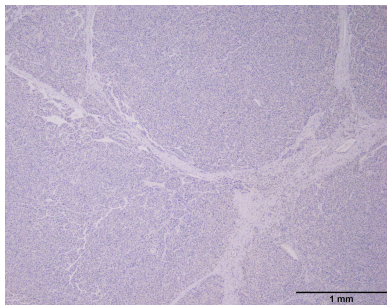

4X

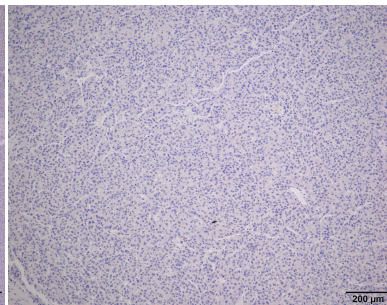

10X

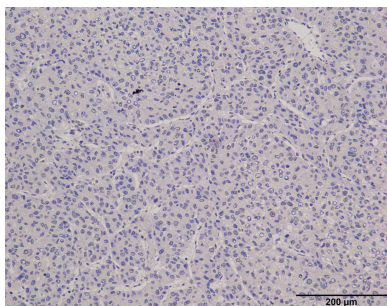

20X

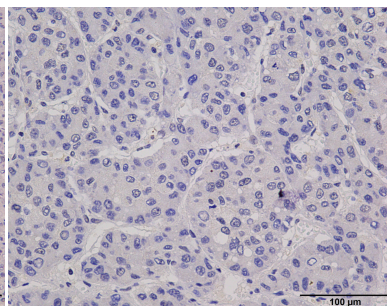

40X

2. P2

N2

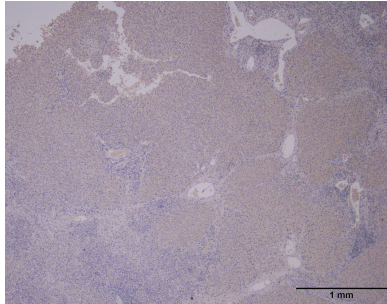

4X

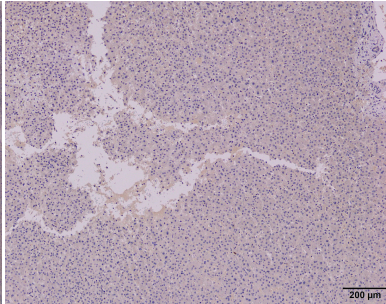

10X

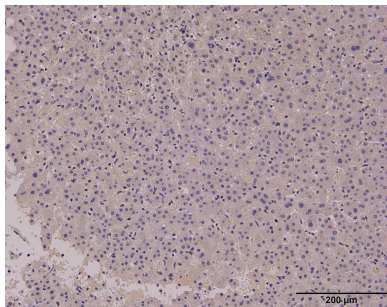

20X

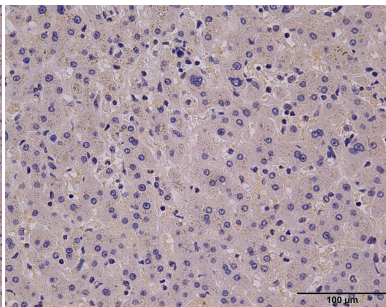

40X

T2

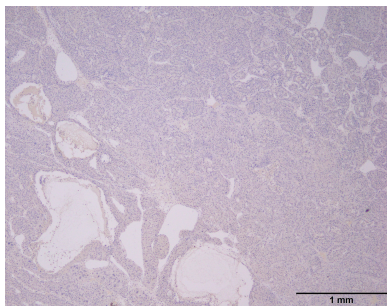

4X

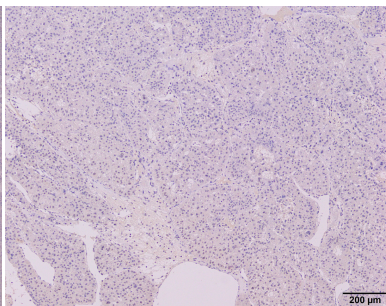

10X

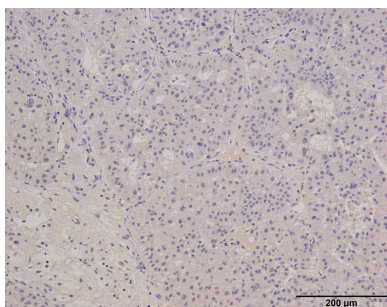

20X

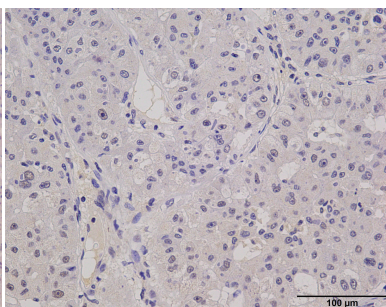

40X

### 3. P3

N3

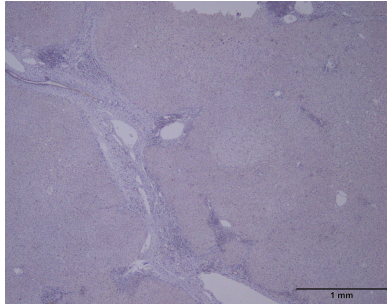

4X

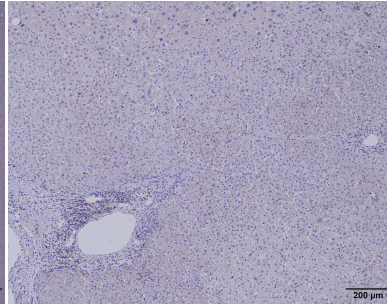

10X

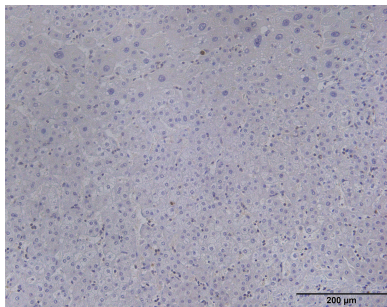

20X

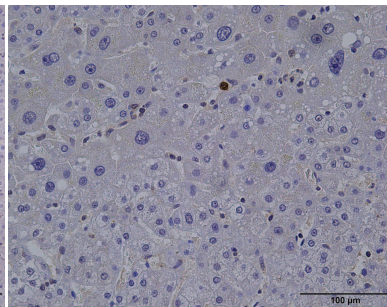

40X

T3

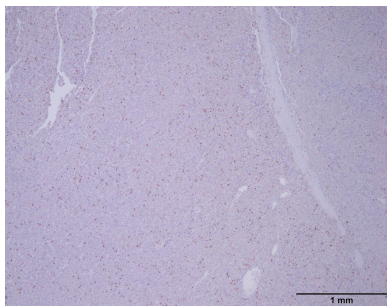

4X

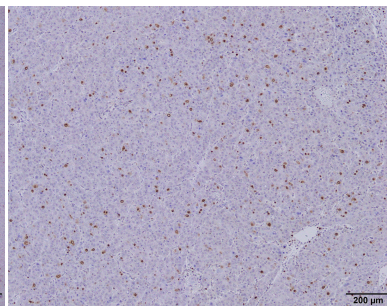

10X

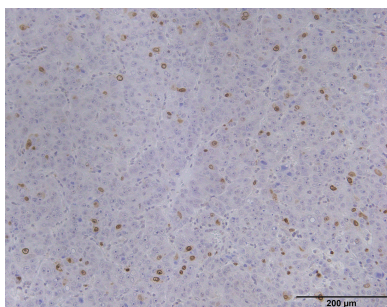

20X

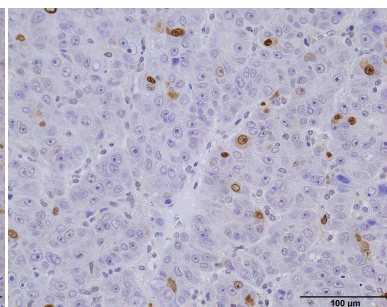

40X

4. P4

N4

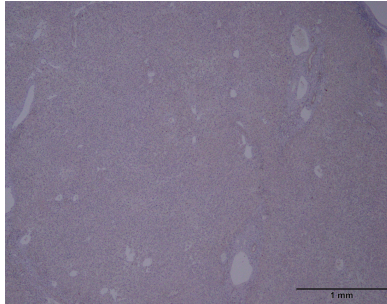

4X

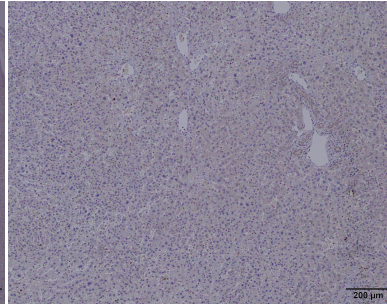

10X

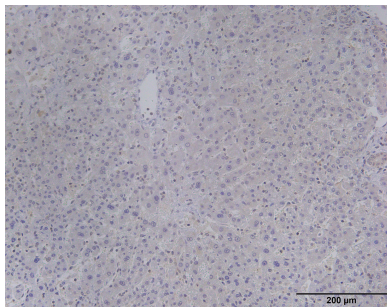

20X

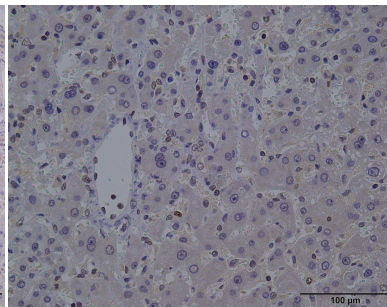

40X

T4

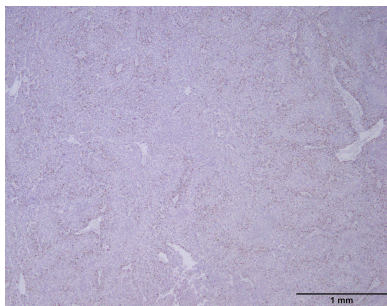

4X

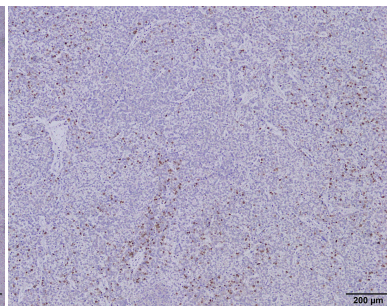

10X

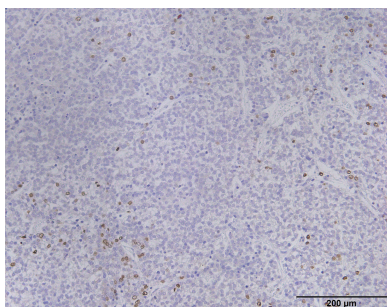

20X

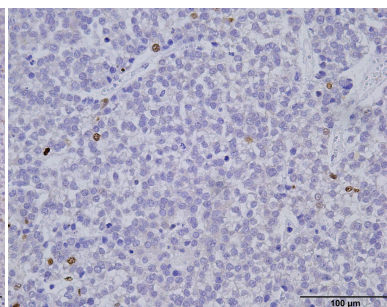

40X

Supplement: Supplementary file 1 — Supplementary Information 1. [file 41598_2024_64907_MOESM1_ESM.pdf]

DEGs\_heatmap

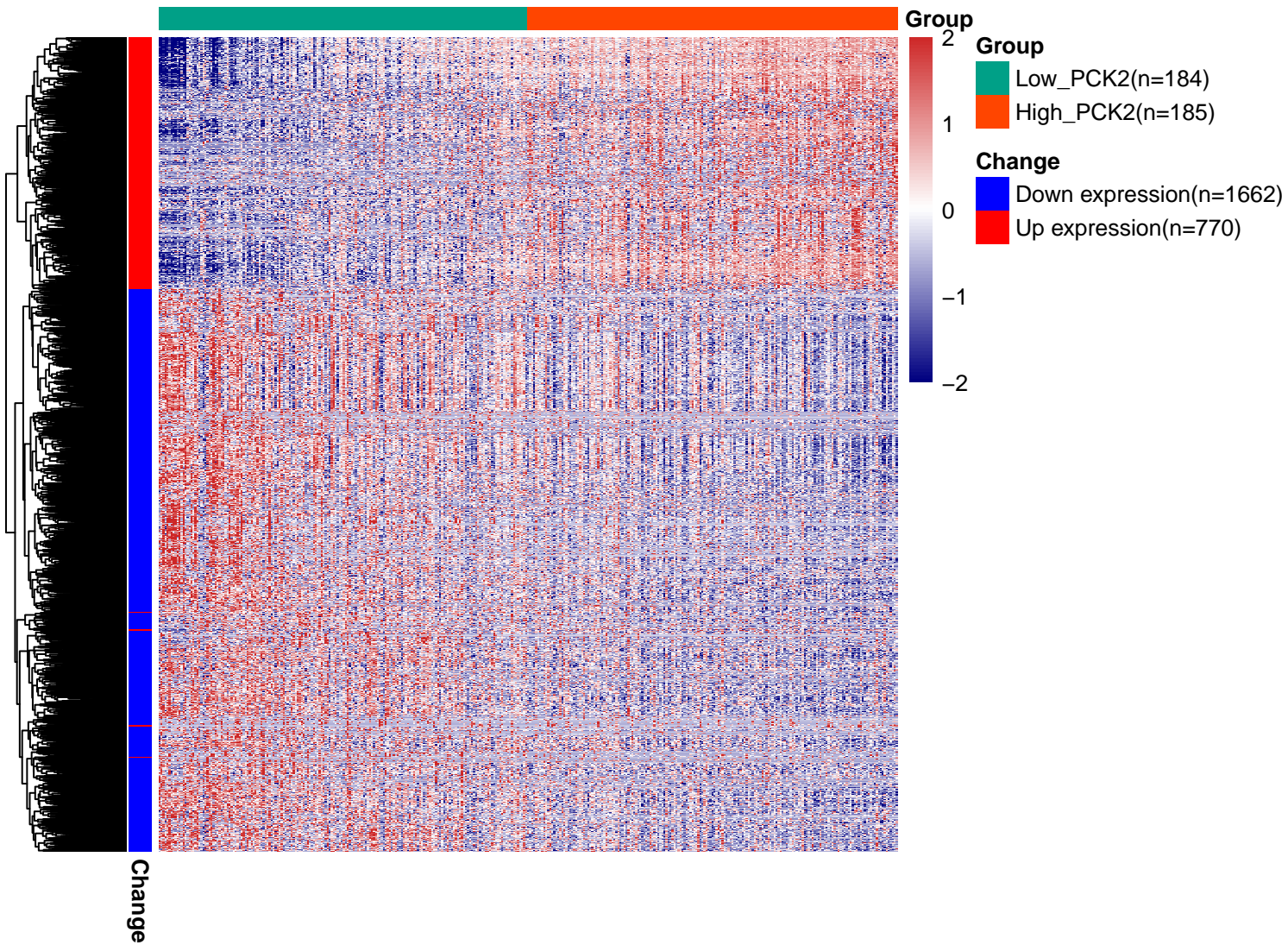

Supplement: Supplementary file 3 — Supplementary Information 3. [file 41598_2024_64907_MOESM3_ESM.pdf]

DEGs\_volcano

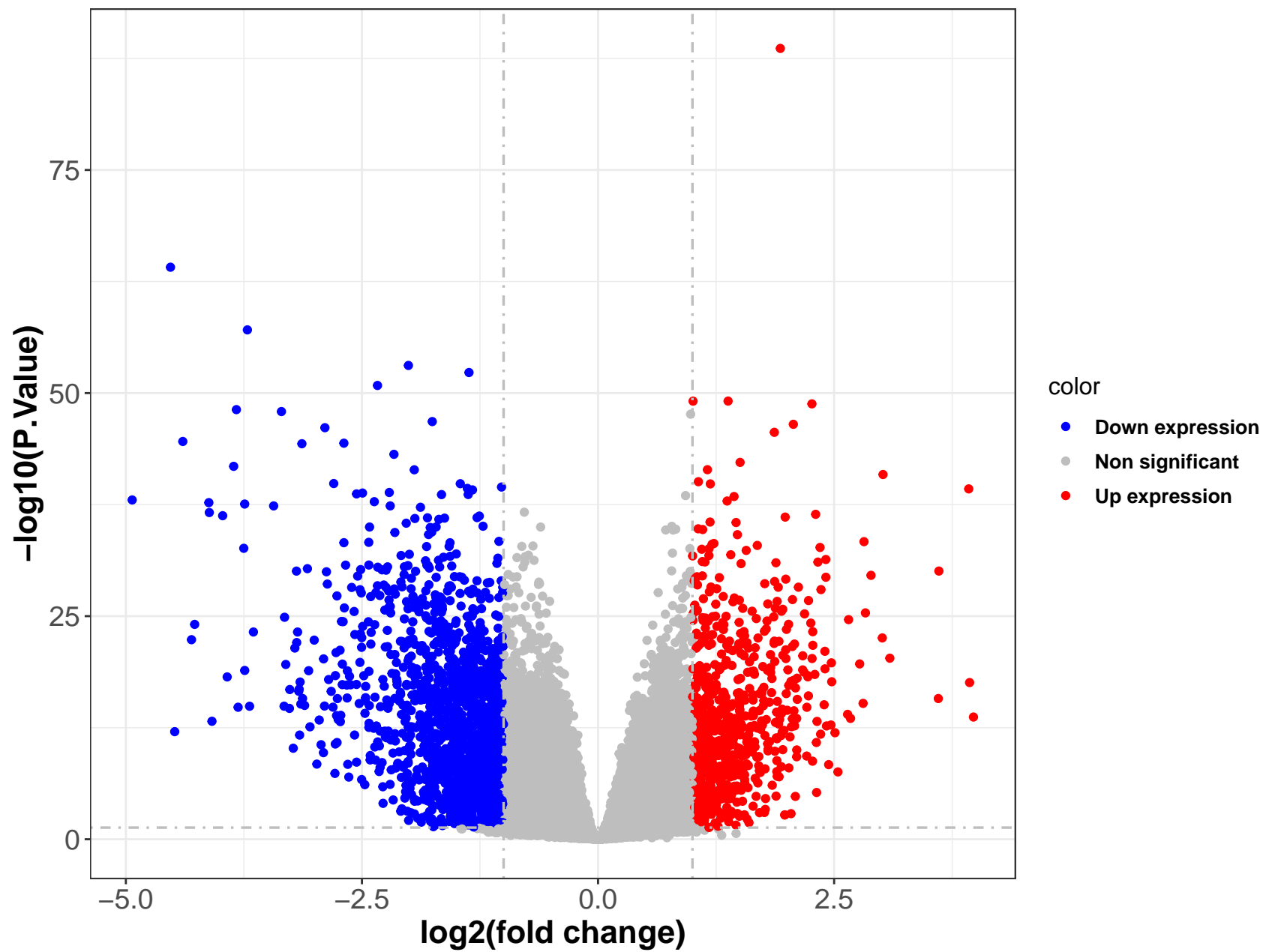

Supplement: Supplementary file 4 — Supplementary Information 4. [file 41598_2024_64907_MOESM4_ESM.pdf]
