# Supplementary material for: Association of mitochondrial phosphoenolpyruvate carboxykinase with prognosis and immune regulation in hepatocellular carcinoma
Source: Sci Rep. 2024 Jun 18;14:14051. doi: 10.1038/s41598-024-64907-7 (PMC11189538; doi:10.1038/s41598-024-64907-7)

Western blot original image

Catalogue

1. Figure1.H 1
    1.1 First experiment (Figure1.H) 2
        P1P2 2
        P3P4 2
        P5P6 2
        P7P8 2
    1.2 Second experiment 3
        P1P2 3
        P3P4 3
        P5P6 3
        P7P8 3
    1.3 Third experiment 4
        P1P2 4
        P3P4 4
        P5P6 4
        P7P8 4
2. Figure10.A 5
    2.1 First experiment 5
    2.2 Second experiment 6
    2.3 Third experiment (Figure10.A) 6
    2.4 Fourth experiment 6
3. Figure10.B 7
    3.1 Figure10.B HepG2 7
        3.1.1 First experiment 7
        3.1.2 Second experiment 8
        3.1.3 Third experiment (Figure10.B HepG2) 8
    3.2 Figure10.B HuH-7 8
        3.2.1 First experiment (Figure10.B HuH-7) 8
        3.2.2 Second experiment 9
        3.2.3 Third experiment 9
4. Figure11.A 10
    4.1 Figure11.A SK-Hep1 10
        4.1.1 First experiment (Figure11.A) 10
        4.1.2 Second experiment 10
        4.1.3 Third experiment 11
    4.2 Figure11.A LM3 11
        4.2.1 First experiment (Figure11.A) 11

4.2.2 Second experiment 12
4.2.3 Third experiment 12
5. Figure12.A 13
5.1 Figure12.A HepG2 13
5.1.1 First experiment (Figure12.A) 13
5.1.2 Second experiment 14
5.1.3 Third experiment 14
5.2 Figure12.A HuH-7 15
5.2.1 First experiment (Figure12.A) 15
5.2.2 Second experiment 15
5.2.3 Third experiment 16
6. Figure12.B 17
6.1 Figure12.B SK-Hep1 17
6.1.1 First experiment (Figure12.B) 17
6.1.2 Second experiment 18
6.1.3 Third experiment 18
6.2 Figure12.B LM3 19
6.2.1 First experiment (Figure12.B) 19
6.2.2 Second experiment 19
6.2.3 Third experiment 20

1. Figure1.H

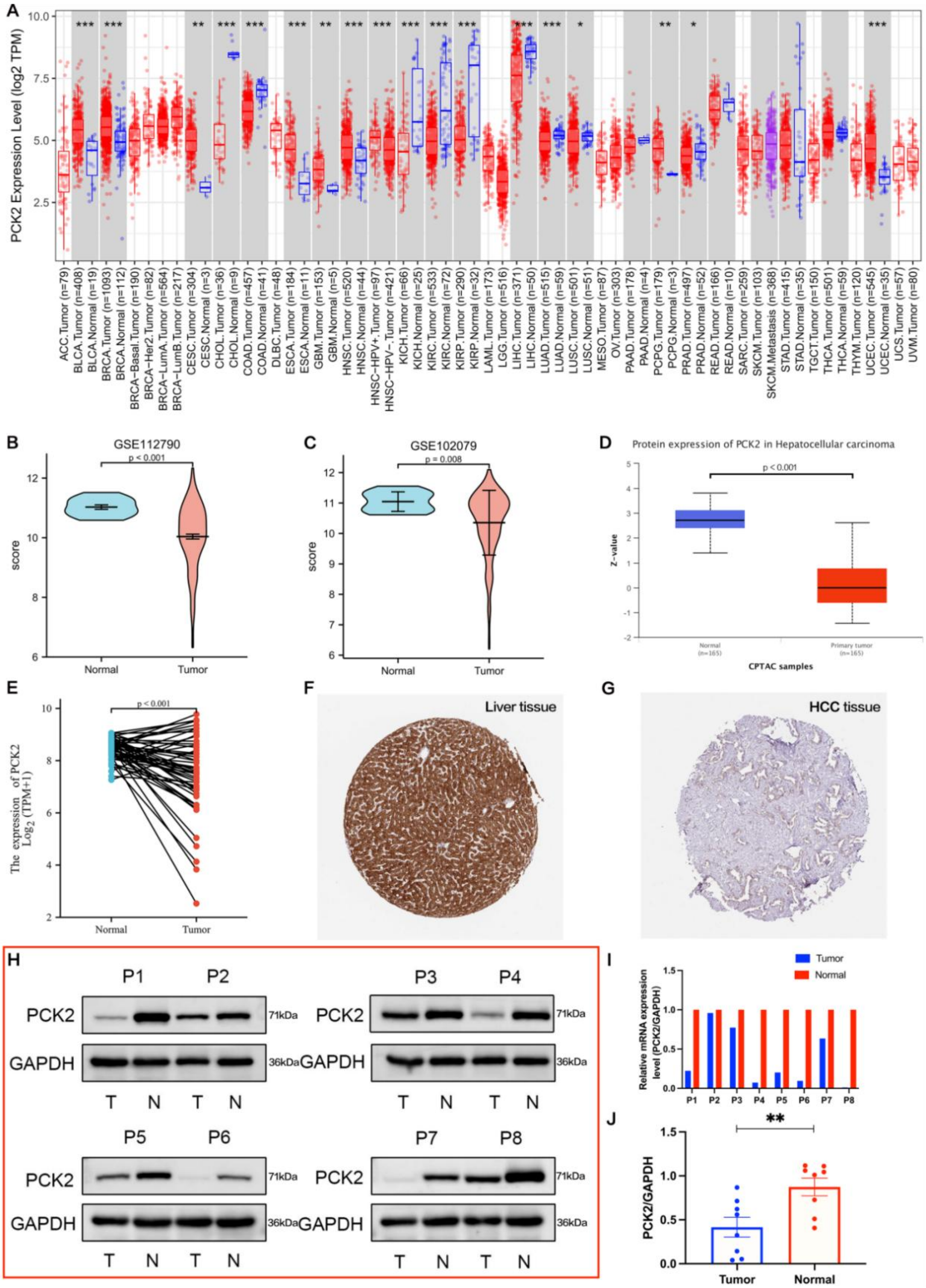
A
PCK2 Expression Level (log2 TPM)
B
GSE112790
p < 0.001
score
Normal
Tumor
C
GSE102079
p = 0.008
score
Normal
Tumor
D
Protein expression of PCK2 in Hepatocellular carcinoma
p < 0.001
Z-value
CPTAC samples
E
p < 0.001
Normal
Tumor
F
Liver tissue
G
HCC tissue
H
P1
P2
P3
P4
P5
P6
P7
P8
PCK2
GAPDH
71kDa
36kDa
T
N
I
Tumor
Normal
Relative mRNA expression level (PCK2/GAPDH)
J
**
PCK2/GAPDH
Tumor
Normal

1.1 First experiment (Figure1.H)

P1P2

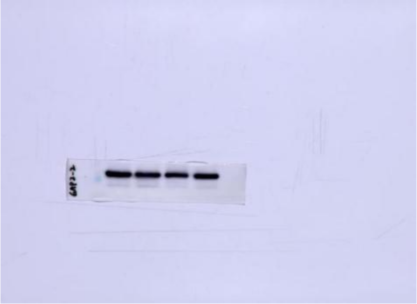

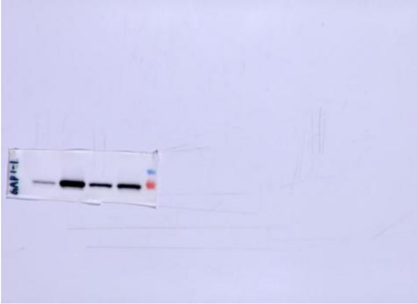

P3P4

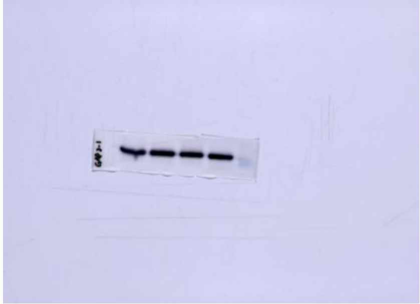

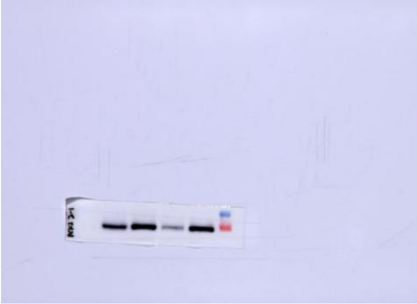

P5P6

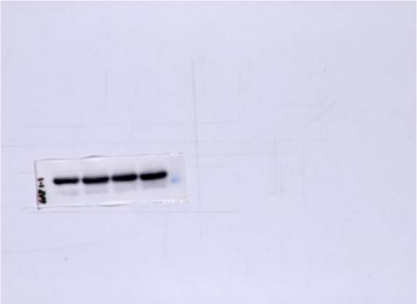

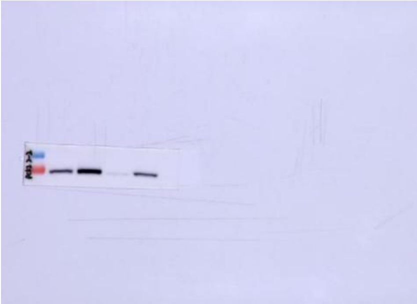

P7P8

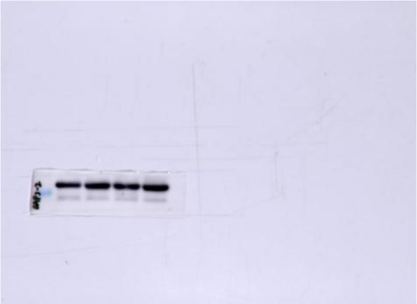

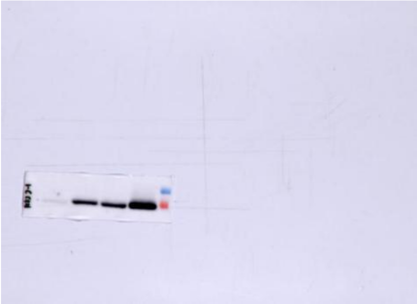

1.2 Second experiment

P1P2

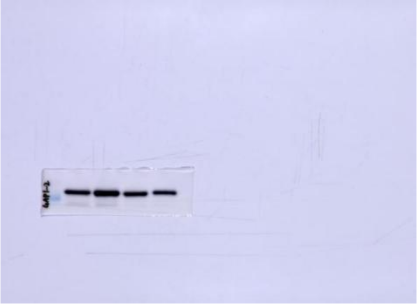

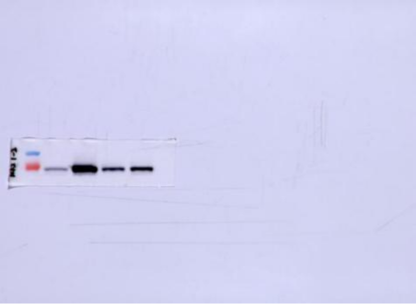

P3P4

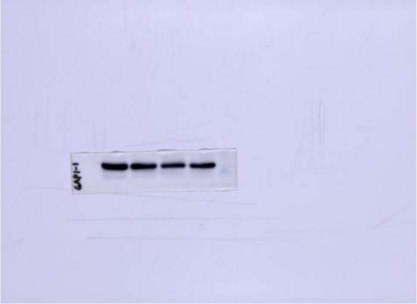

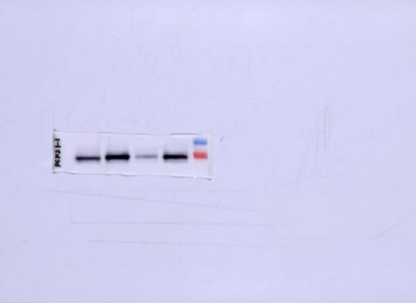

P5P6

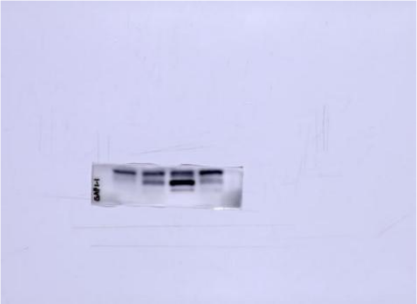

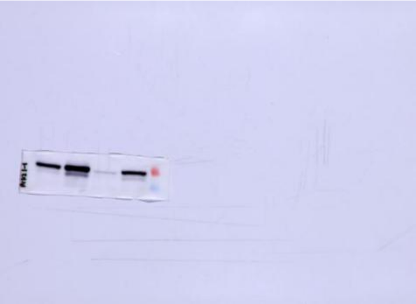

P7P8

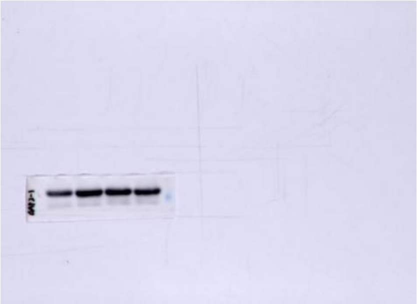

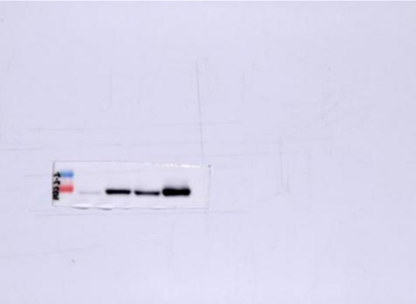

1.3 Third experiment

P1P2

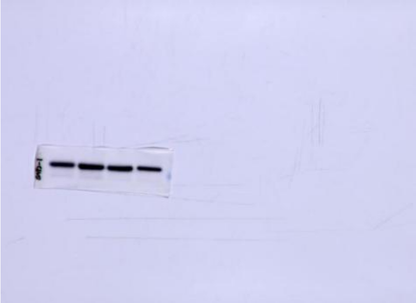
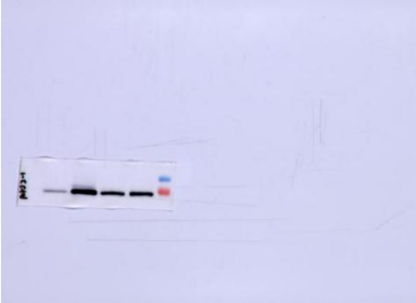

P3P4

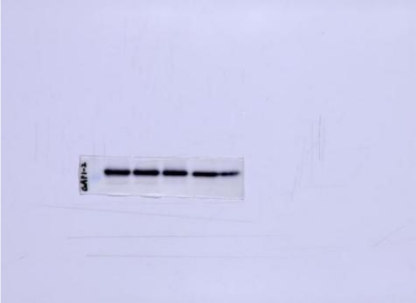
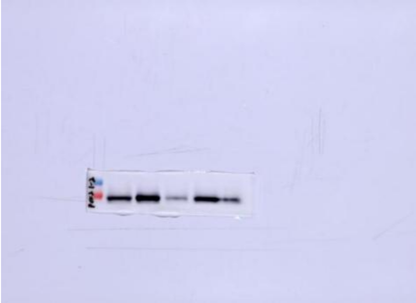

P5P6

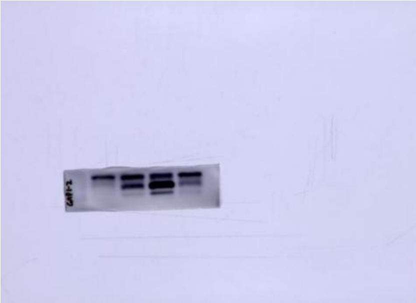
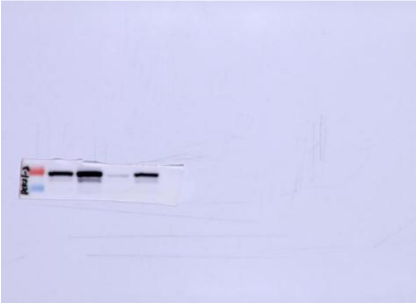

P7P8

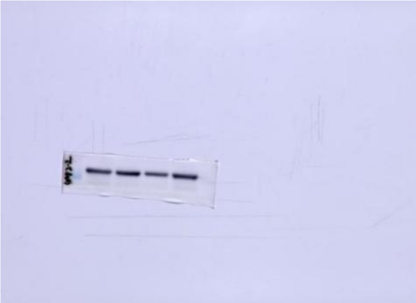
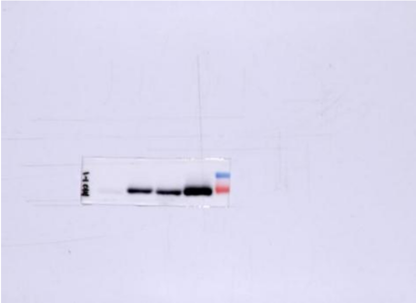

2. Figure10.A

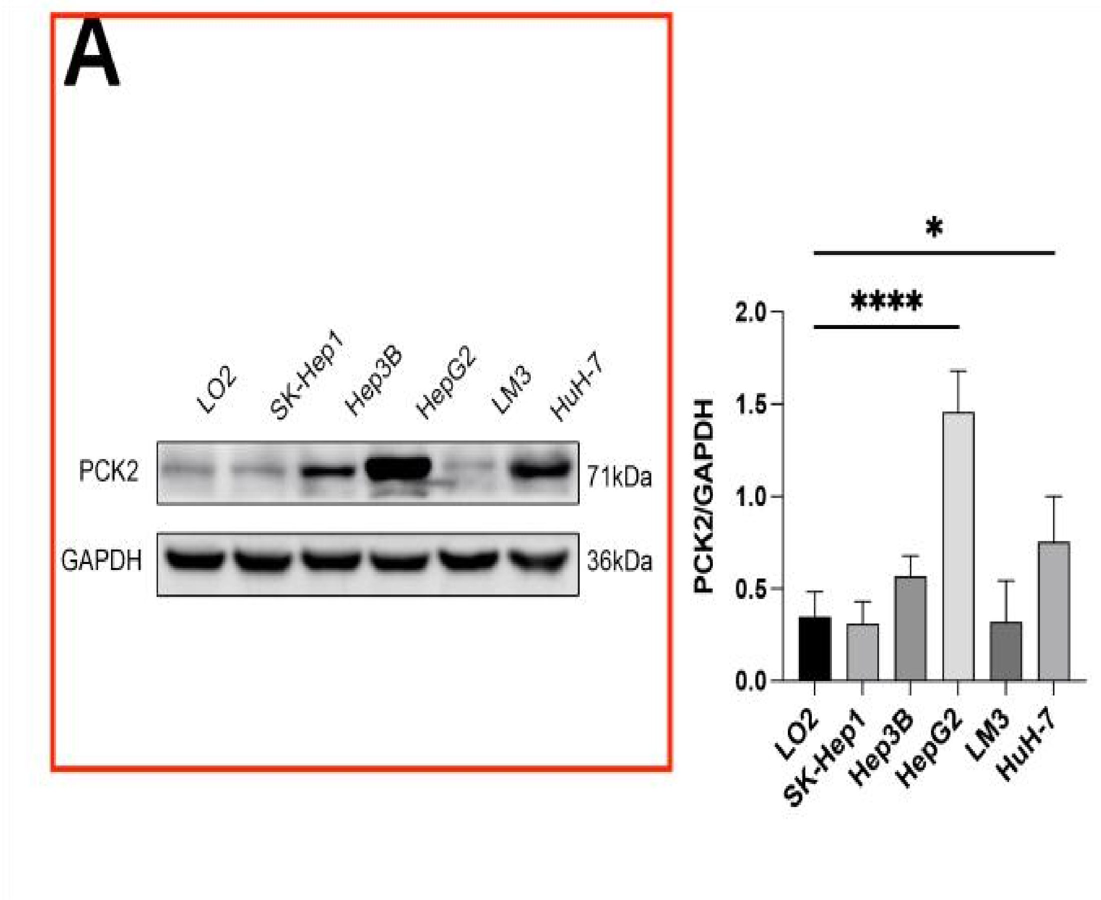

2.1 First experiment

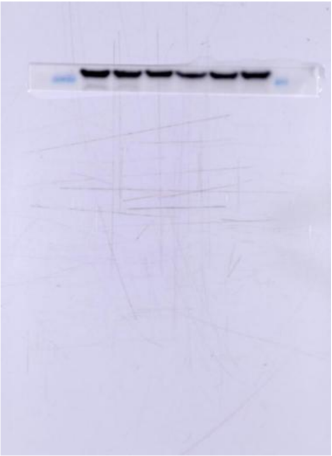
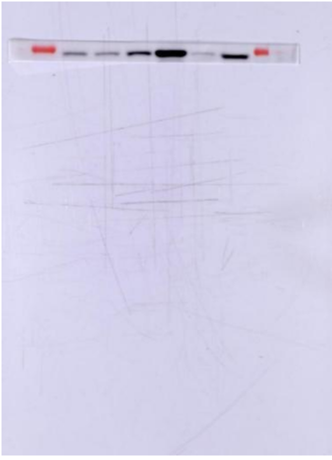

2.2 Second experiment

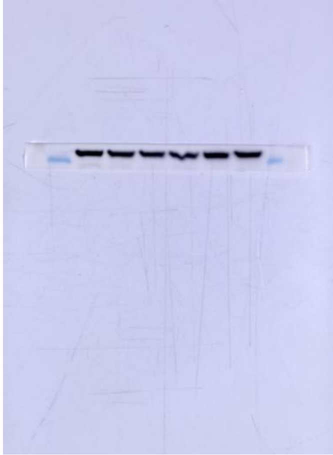

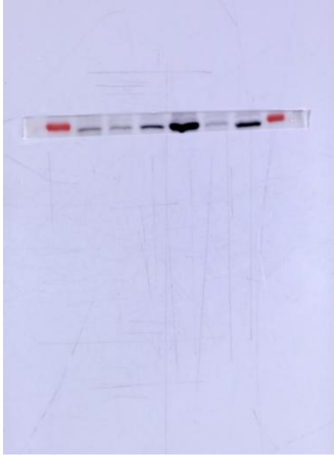

2.3 Third experiment (Figure10.A)

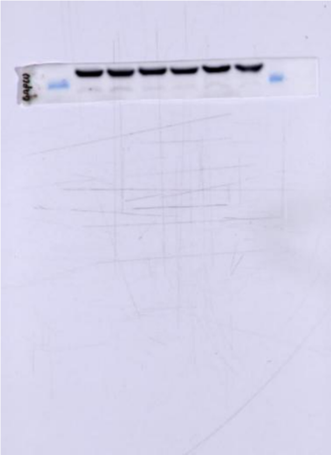

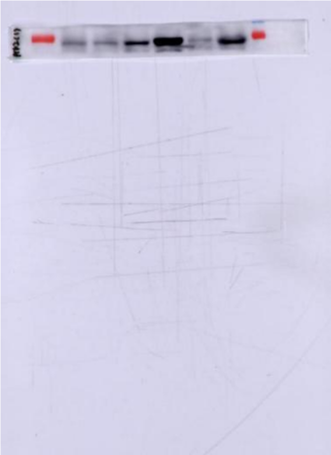

2.4 Fourth experiment

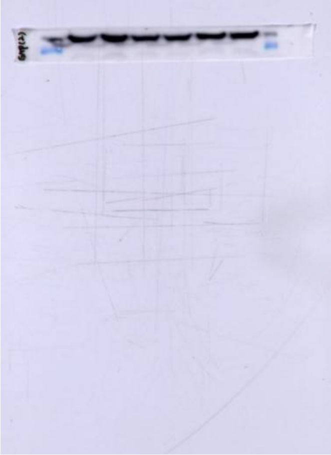

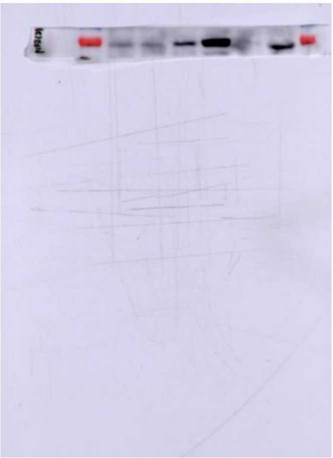

3. Figure10.B

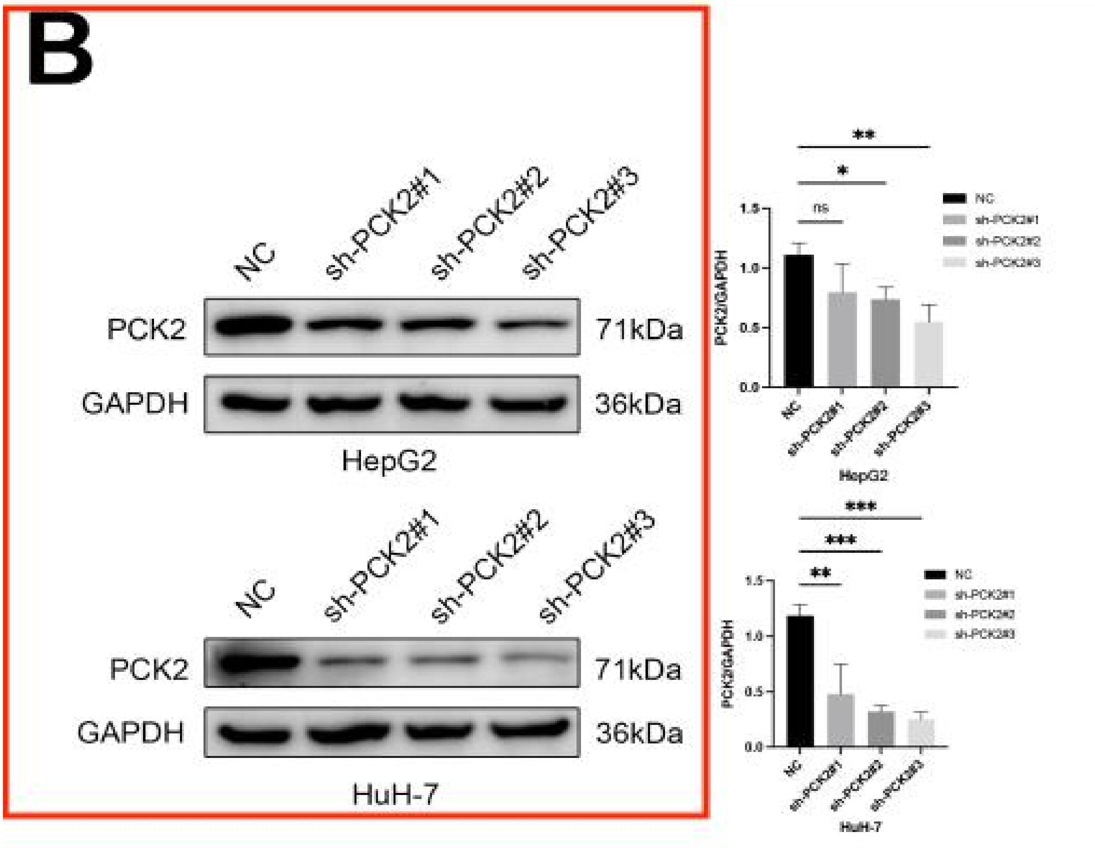

3.1 Figure10.B HepG2

3.1.1 First experiment

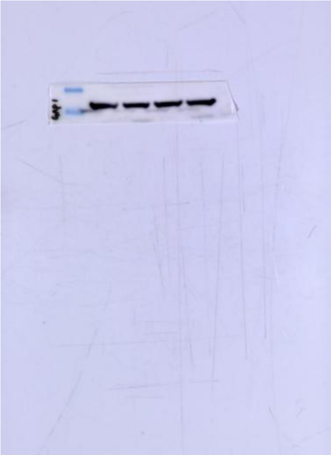

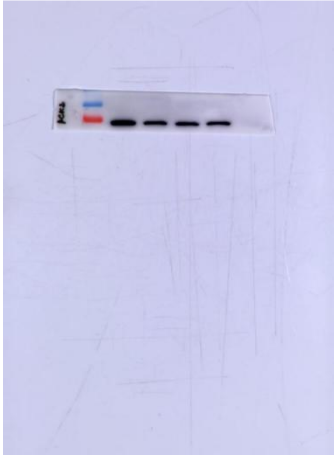

3.1.2 Second experiment

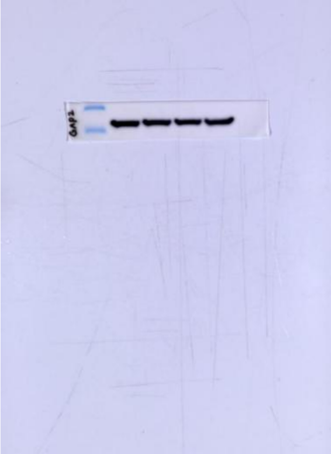

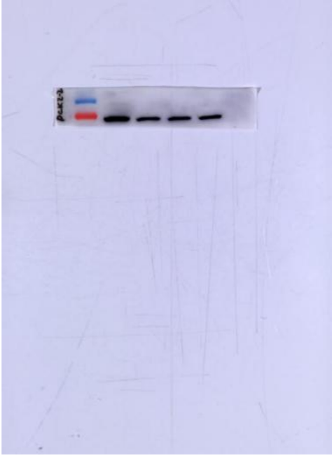

3.1.3 Third experiment (Figure10.B HepG2)

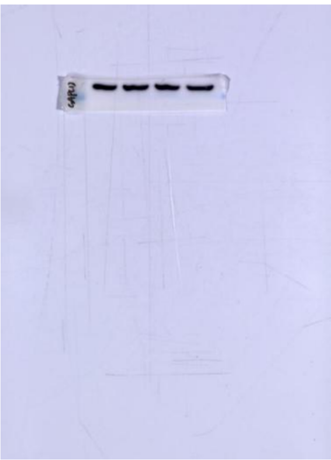

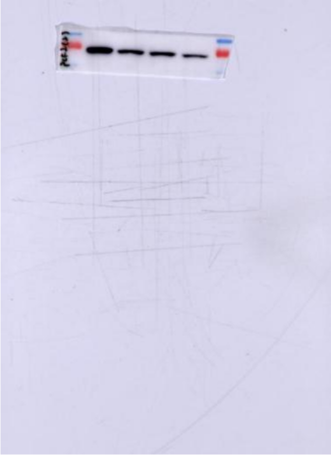

3.2 Figure10.B HuH-7

3.2.1 First experiment (Figure10.B HuH-7)

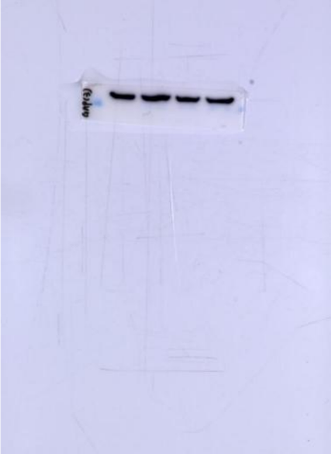

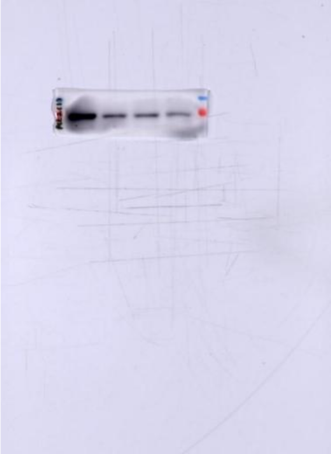

3.2.2 Second experiment

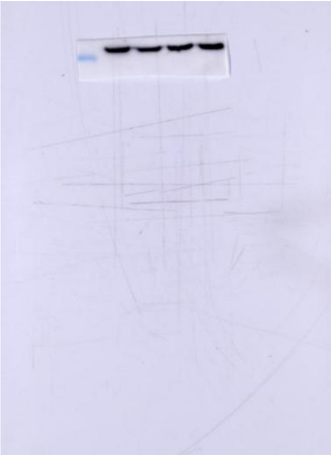

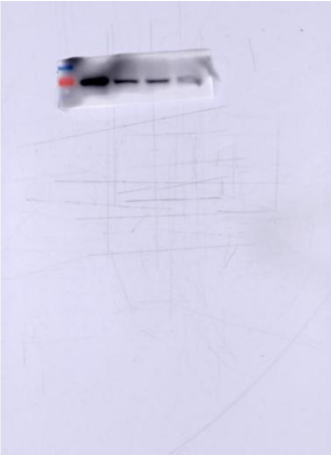

3.2.3 Third experiment

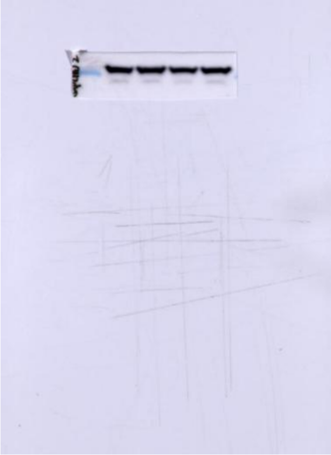

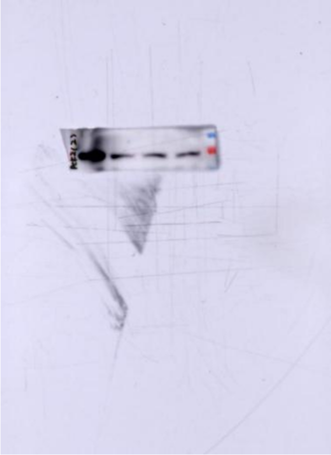

4. Figure11.A

4.1 Figure11.A SK-Hep1

4.1.1 First experiment (Figure11.A)

Vector

PCK2

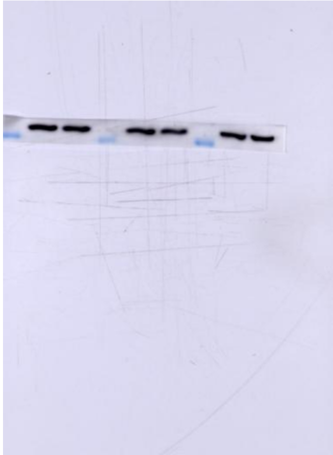

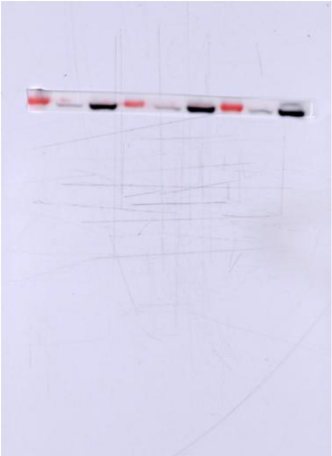

4.1.2 Second experiment

Vector

PCK2

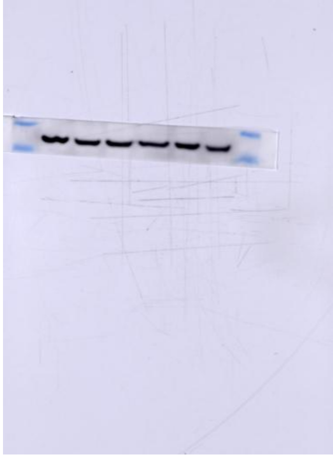

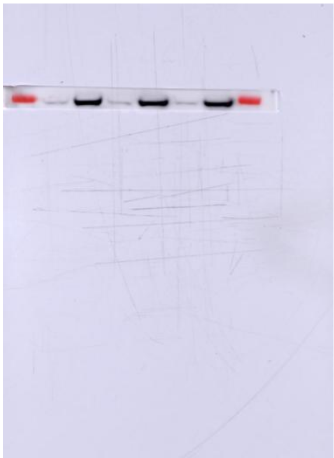

4.1.3 Third experiment

Vector

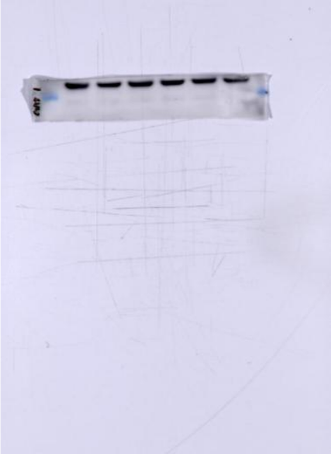

PCK2

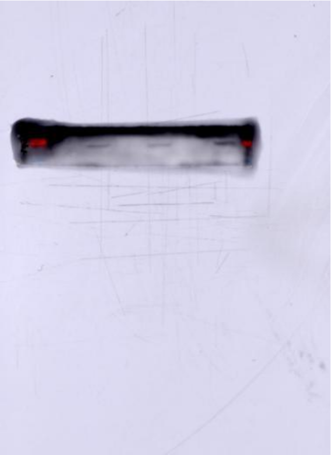

4.2 Figure11.A LM3

4.2.1 First experiment (Figure11.A)

Vector

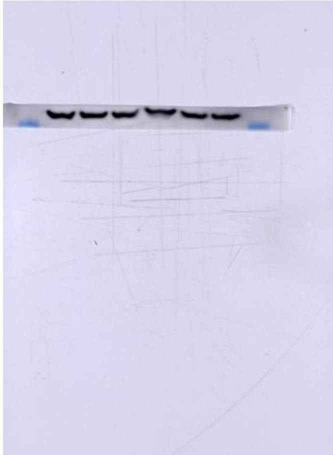

PCK2

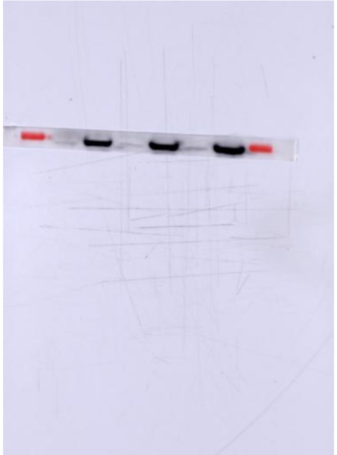

4.2.2 Second experiment

Vector PCK2

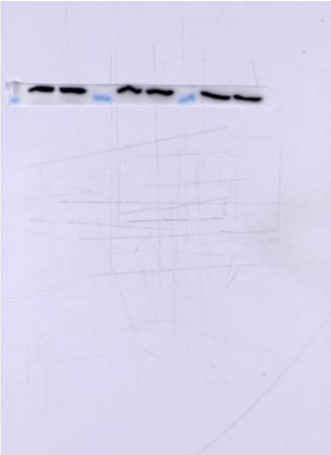

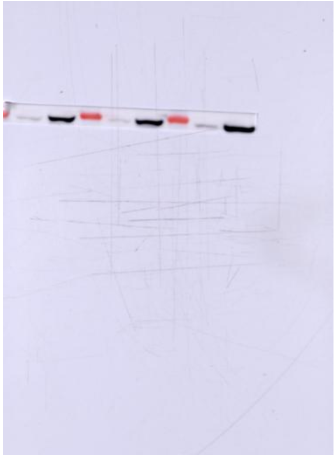

4.2.3 Third experiment

Vector PCK2

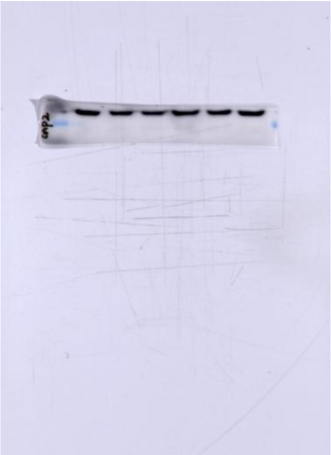

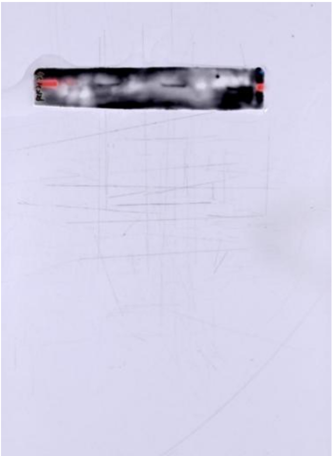

5. Figure12.A

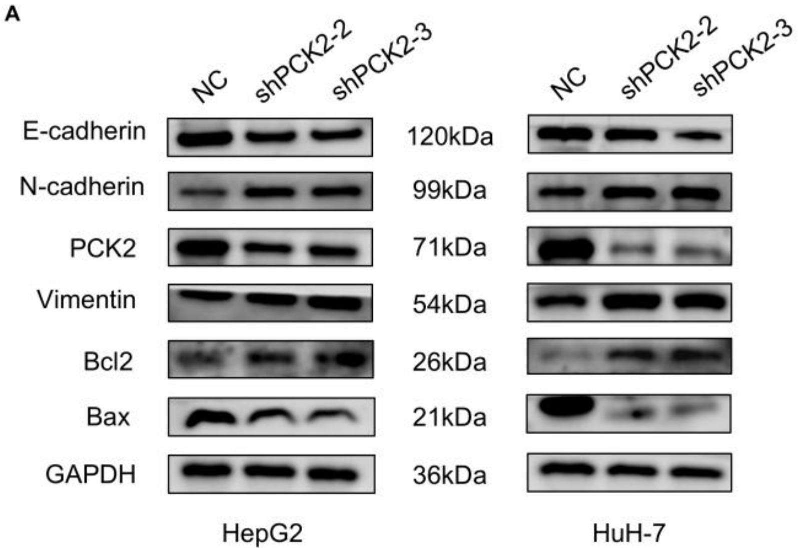

5.1 Figure12.A HepG2

5.1.1 First experiment (Figure12.A)

E-cadherin

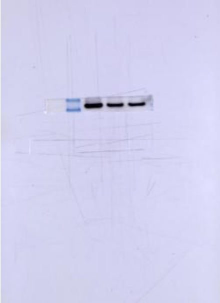

N-cadherin

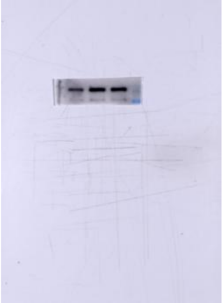

PCK2

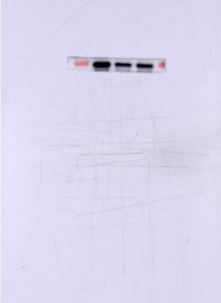

Vimentin

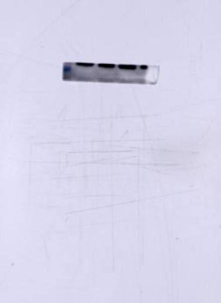

Bcl2

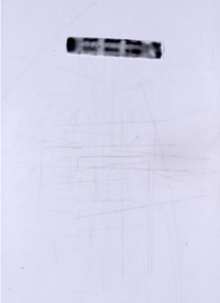

Bax

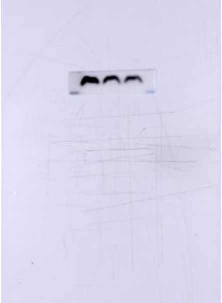

GAPDH

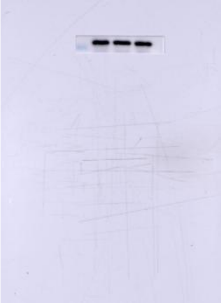

5.1.2 Second experiment

E-cadherin

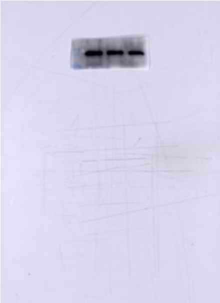

N-cadherin

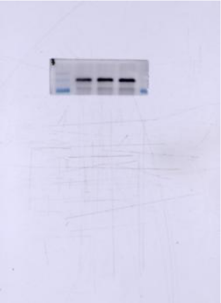

PCK2

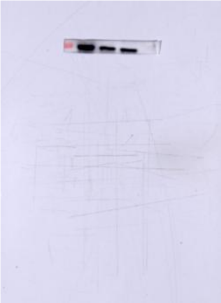

Vimentin

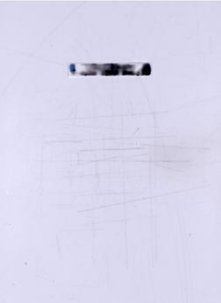

Bcl2

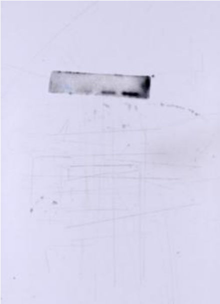

Bax

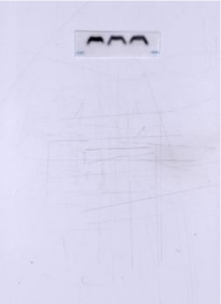

GAPDH

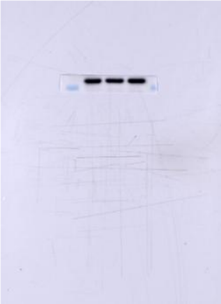

5.1.3 Third experiment

E-cadherin

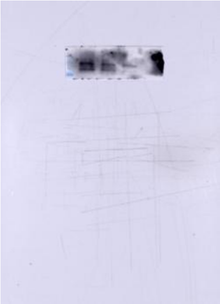

N-cadherin

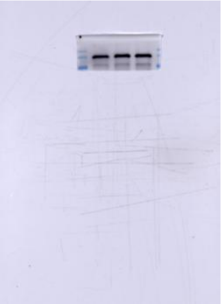

PCK2

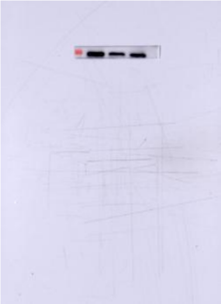

Vimentin

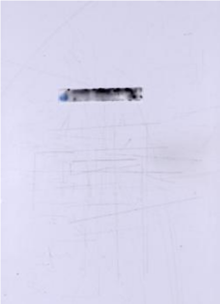

Bcl2

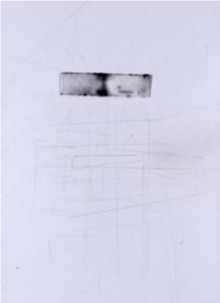

Bax

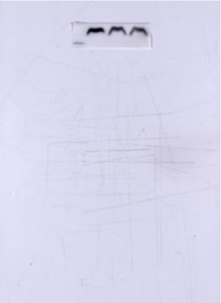

GAPDH

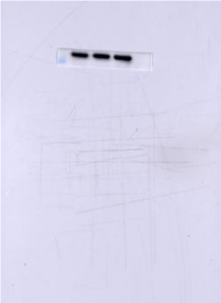

5.2 Figure12.A HuH-7

5.2.1 First experiment (Figure12.A)

E-cadherin

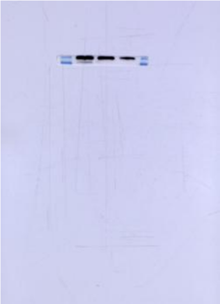

N-cadherin

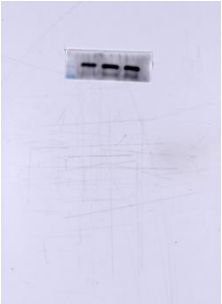

PCK2

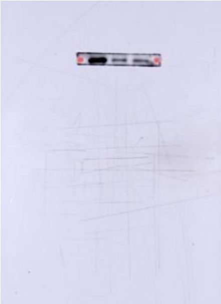

Vimentin

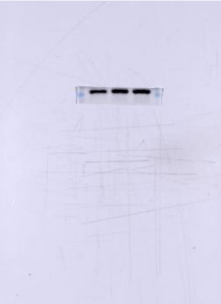

Bcl2

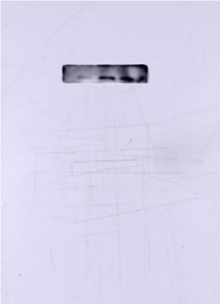

Bax

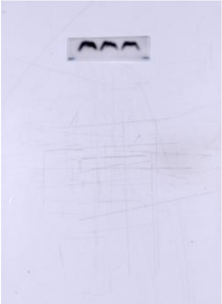

GAPDH

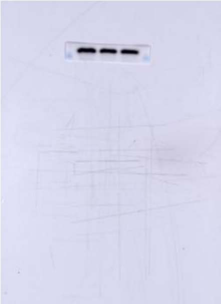

5.2.2 Second experiment

E-cadherin

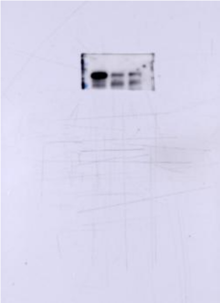

N-cadherin

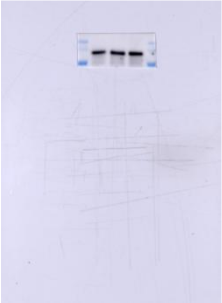

PCK2

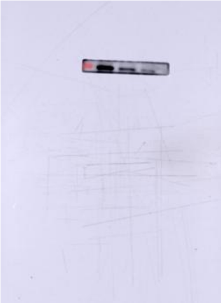

Vimentin

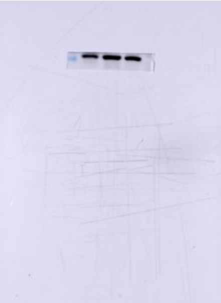

Bcl2

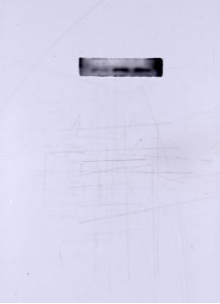

Bax

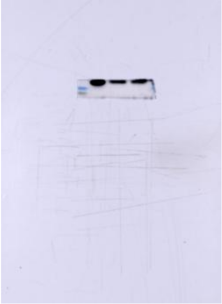

GAPDH

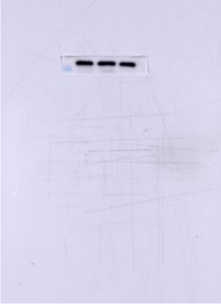

5.2.3 Third experiment

E-cadherin

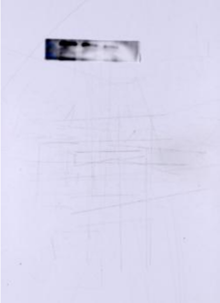

N-cadherin

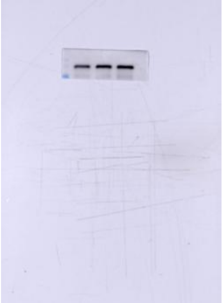

PCK2

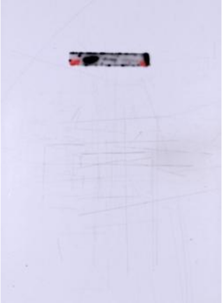

Vimentin

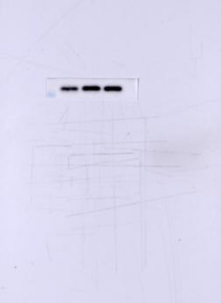

Bcl2

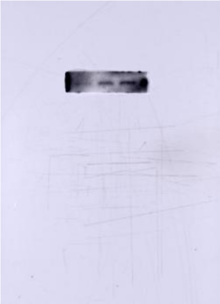

Bax

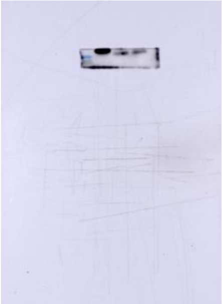

GAPDH

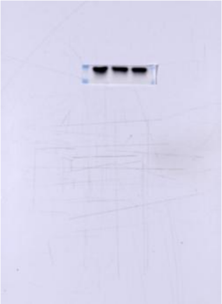

6. Figure12.B

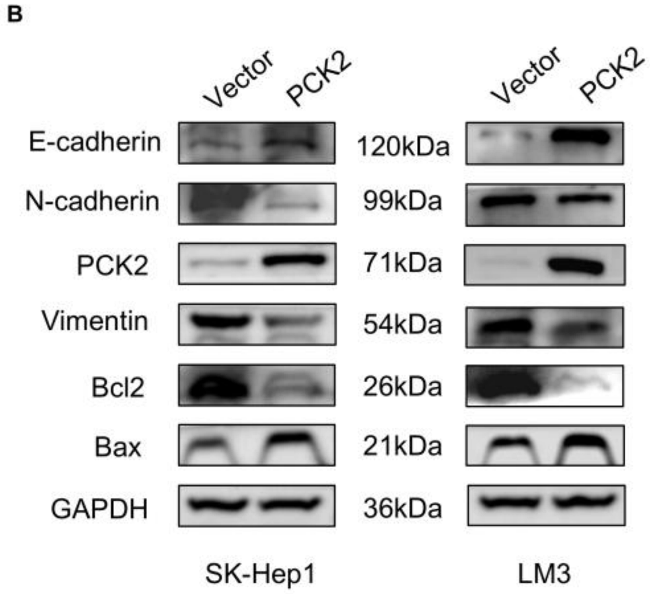

6.1 Figure12.B SK-Hep1

6.1.1 First experiment (Figure12.B)

E-cadherin

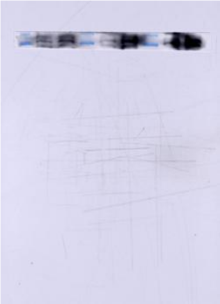

N-cadherin

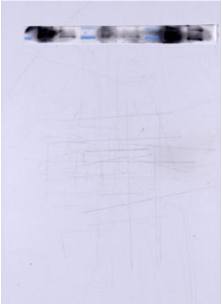

PCK2

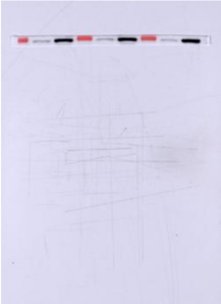

Vimentin

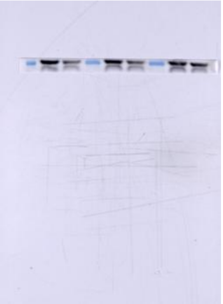

Bcl2

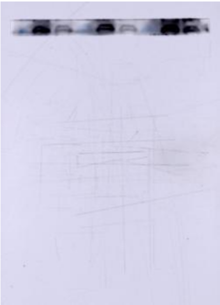

Bax

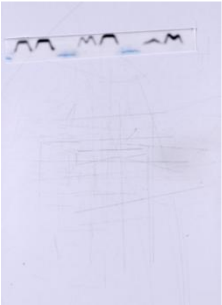

GAPDH

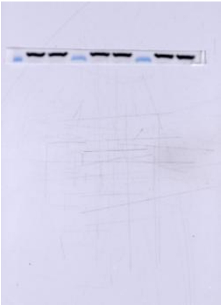

6.1.2 Second experiment

E-cadherin

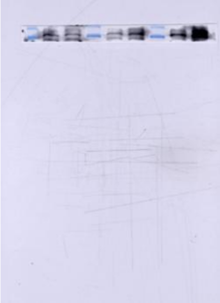

N-cadherin

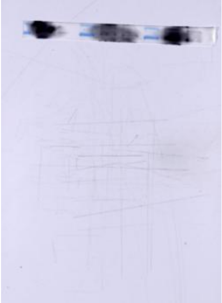

PCK2

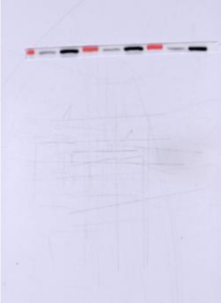

Vimentin

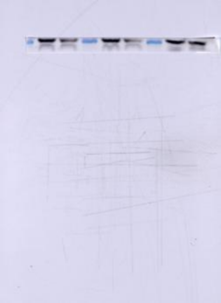

Bcl2

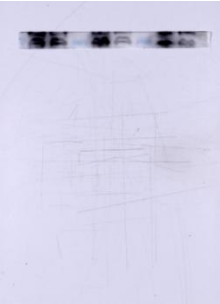

Bax

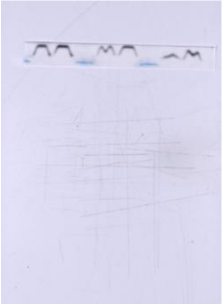

GAPDH

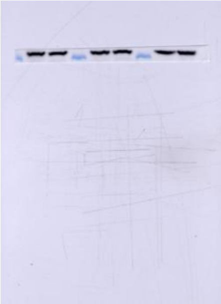

6.1.3 Third experiment

E-cadherin

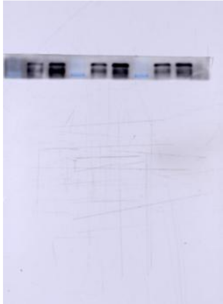

N-cadherin

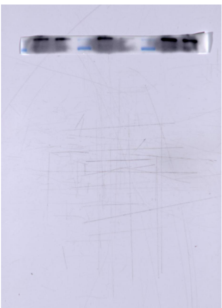

PCK2

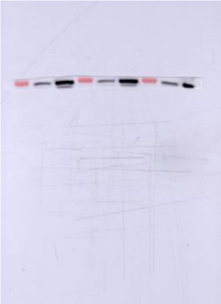

Vimentin

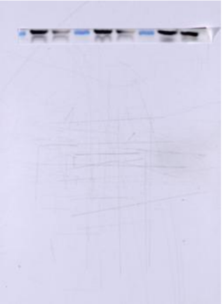

Bcl2

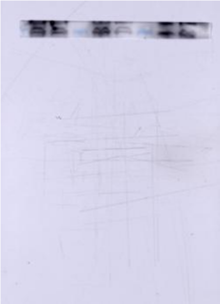

Bax

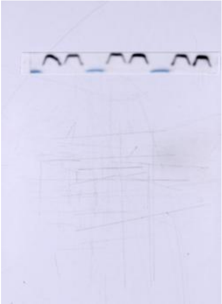

GAPDH

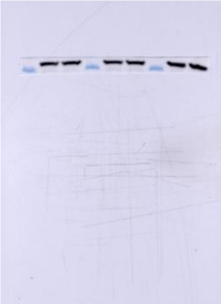

6.2 Figure12.B LM3

6.2.1 First experiment (Figure12.B)

E-cadherin

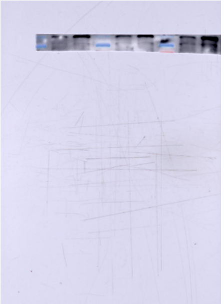

N-cadherin

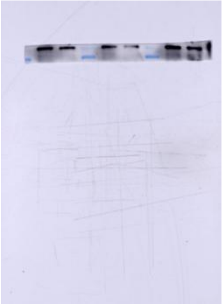

PCK2

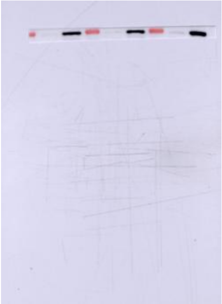

Vimentin

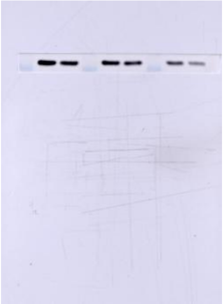

Bcl2

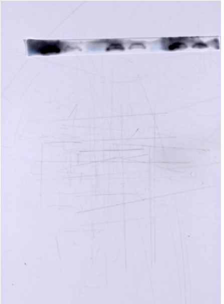

Bax

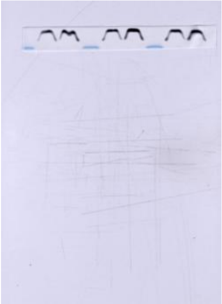

GAPDH

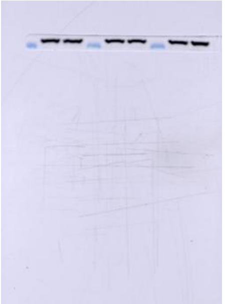

6.2.2 Second experiment

E-cadherin

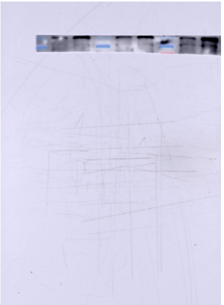

N-cadherin

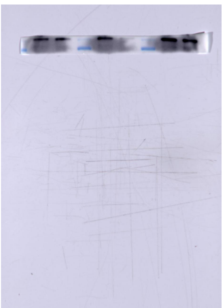

PCK2

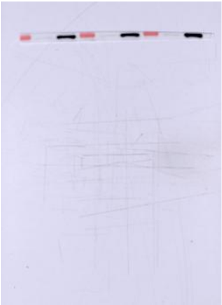

Vimentin

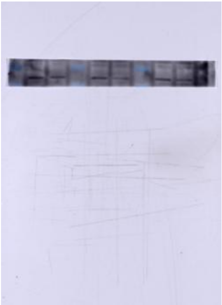

Bcl2

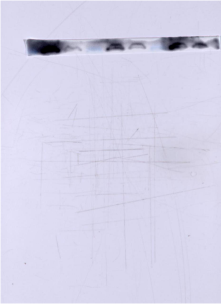

Bax

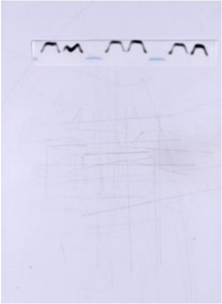

GAPDH

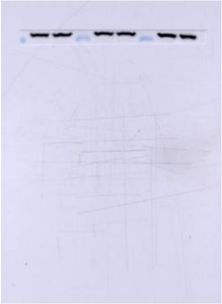

6.2.3 Third experiment

E-cadherin

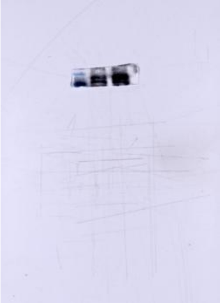

N-cadherin

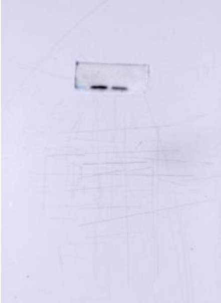

PCK2

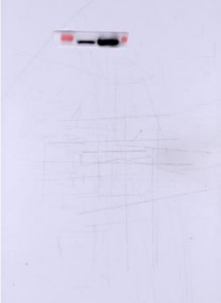

Vimentin

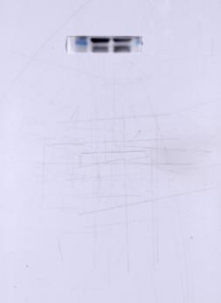

Bcl2

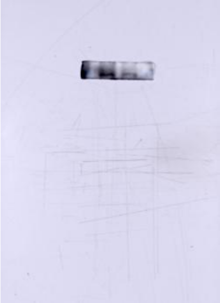

Bax

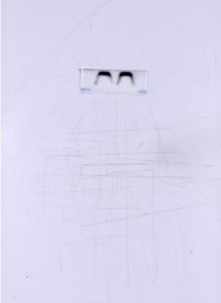

GAPDH

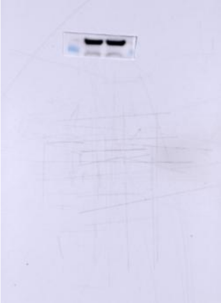

Supplement: Supplementary file 12 — Supplementary Information 12. [file 41598_2024_64907_MOESM12_ESM.pdf]
